# Insights into structural, spectroscopic, and hydrogen bonding interaction patterns of nicotinamide–oxalic acid (form I) salt by using experimental and theoretical approaches

**DOI:** 10.3389/fchem.2023.1203278

**Published:** 2023-07-05

**Authors:** Priya Verma, Anubha Srivastava, Poonam Tandon, Manishkumar R. Shimpi

**Affiliations:** ^1^ Department of Physics, University of Lucknow, Lucknow, India; ^2^ Department of Materials and Environmental Chemistry, Stockholm University, Stockholm, Sweden

**Keywords:** nicotinamide, oxalic acid, NIC-OXA (form I) salt, characterization, spectroscopy, density functional theory, reactivity

## Abstract

In the present work, nicotinamide–oxalic acid (NIC-OXA, form I) salt was crystallized by slow evaporation of an aqueous solution. To understand the molecular structure and spectroscopic properties of NIC after co-crystallization with OXA, experimental infrared (IR), Raman spectroscopic signatures, X-ray powder diffraction (XRPD), and differential scanning calorimetry (DSC) techniques were used to characterize and validate the salt. The density functional theory (DFT) methodology was adopted to perform all theoretical calculations by using the B3LYP/6-311++G (d, p) functional/basis set. The experimental geometrical parameters were matched in good correlation with the theoretical parameters of the dimer than the monomer, due to the fact of covering the nearest hydrogen bonding interactions present in the crystal structure of the salt. The IR and Raman spectra of the dimer showed the red (downward) shifting and broadening of bands among (N15-H16), (N38-H39), and (C13=O14) bonds of NIC and (C26=O24), (C3=O1), and (C26=O25) groups of OXA, hence involved in the formation of NIC-OXA salt. The atoms in molecules (AIM) analysis revealed that (N8-H9**···**O24) is the strongest (conventional) intermolecular hydrogen bonding interaction in the dimer model of salt with the maximum value of interaction energy −12.1 kcal mol^−1^. Furthermore, the natural bond orbital (NBO) analysis of the Fock matrix showed that in the dimer model, the (N8-H9···O24) bond is responsible for the stabilization of the salt with an energy value of 13.44 kcal mol^−1^. The frontier molecular orbitals (FMOs) analysis showed that NIC-OXA (form I) salt is more reactive and less stable than NIC, as the energy gap of NIC-OXA (form I) salt is less than that of NIC. The global and local reactivity descriptor parameters were calculated for the monomer and dimer models of the salt. The electrophilic, nucleophilic, and neutral reactive sites of NIC, OXA, monomer, and dimer models of salt were visualized by plotting the molecular electrostatic potential (MESP) surface. The study provides valuable insights into combining both experimental and theoretical results that could define the physicochemical properties of molecules.

## 1 Introduction

Pepinsky proposed the term “crystal engineering” to denote the process of constructing crystalline materials from molecules or ions using non-covalent synthesis that intended, nowadays, to evaluate or manipulate the structure–property relationship (Pepinsky, 1955; Desiraju, 1989; Bhattacharya et al., 2018). This approach is also applicable in pharmaceutical science, as the active pharmaceutical ingredients (APIs) possess various hydrogen bond possibilities (generally responsible for biological activity) (Desiraju, 1995; Desiraju, 2010; Duggirala et al., 2015). The physicochemical properties of APIs, such as mechanical properties and thermal and chemical stability, can be enhanced or altered with the formation of salts, polymorphs, solvates, and cocrystals (Kumar, 2018; Kumar et al., 2018). Pharmaceutical salt is defined as an ionizable drug that can be formed by complete proton transfer between an API and a coformer (Bhogala et al., 2005; Childs et al., 2007). Salts and cocrystals both can be formed by various non-covalent interactions, such as ionic interactions, π–π stacking, hydrogen bonding, and van der Waals forces (Martin et al., 2013; Steed, 2013; Wang et al., 2015; Chen et al., 2016). Salt formation in crystal engineering is used to modify the drug product with the chemical modification in formulation, development, and biopharmaceutical and therapeutic properties without altering the chemical structure and pharmacology. The concept of crystal engineering is to understand the basic hydrogen bonding networks to discuss the relationship between the molecular and supramolecular structure (Moulton and Zaworotko, 2001; Forni and Pilati, 2002; Shan and Zaworotko, 2008). It is reported that more than 60% of drugs failed in their clinical trials due to low solubility, due to increased molecular weight and lipophilicity (Sareen et al., 2012). Hence, drugs’ development and efficacy have been a popular topic of interest among various scientists and researchers to study the supramolecular structure of drugs with their enhanced properties by using crystal engineering strategies (Vioglio et al., 2017). It has also been demonstrated that the co-crystallization process of drugs can significantly improve the different physicochemical properties of APIs (paracetamol (Srivastava et al., 2019), gabapentin (Wenger and Bernstein, 2008), enoxacin (Liu et al., 2020), carvedilol (Hiendrawan et al., 2017), and so on).

Nicotinamide (water-soluble vitamin B-3; NIC) has attracted a lot of attention from researchers, as it is a cofactor and precursor of nicotinamide adenine dinucleotide (NAD), which is responsible for cell function, growth, survival, and metabolism (Soma and Lalam, 2022). NIC is an orally active form of NAD that can be obtained from plant and animal sources such as broccoli, beef, cucumber, and cow’s milk and can be used to treat pellagra, diabetes, and dietary- and aging-induced obesity (Mills et al., 2016). NIC has proven to be potentially safe and tolerable in humans (Irie et al., 2020; Liao et al., 2021). Oxalic acid (OXA) is used in pharmaceutical salt preparation, and it has shown good tolerability and low toxicity (Benito et al., 2022a). Its main sources are plants, vegetables, fruits, nuts, and seeds (Chukwuebuk and Chinenye, 2015). OXA consists of two carbonyl (C=O) and hydroxyl (OH) groups, in which the (C=O) group can form hydrogen bonds with a proton donor, whereas the OH group can form hydrogen bonds with a proton acceptor, and so can form salts or cocrystals easily (Benito et al., 2022b). The titled salt formation occurs when a proton from OXA is transferred into the nitrogen atom of the pyridine ring of NIC.

In continuation to the previously published work of nicotinamide–oxalic acid (form II) salt (Verma et al., 2022) and nicotinamide–citric acid cocrystal (Verma et al., 2019), in the present study, the characterization, spectroscopic analysis, thermal analysis, hydrogen bond motifs, and reactivity analysis of NIC-OXA (form I) salt were performed. For this purpose, we adopted a combined experimental and density functional theory (DFT) methodology to describe the structural and physicochemical properties of monomer and dimer models of NIC-OXA (form I) salt. The molecular structures and spectra were obtained by B3LYP functional employing the 6-311++G (d,p) basis set and compared with the experimental results to get an insight into the pattern of API (NIC) before and after its salt formation with the coformer (OXA). The X-ray powder diffraction (XRPD) (Spiliopoulou et al., 2020) pattern was obtained to discuss the phase composition of the titled salt. The differential scanning calorimetry (DSC) (Amarachi, 2020) technique was used to study the thermal behavior and evaluate the traces of the physiosorbed water molecules. The AIM and NBO analyses proved the nature and strength of hydrogen bonding present in salt, respectively. Furthermore, global and local reactivity descriptors have been calculated to examine the chemical properties of salt. This work sheds light on the geometrical and physicochemical properties of NIC-OXA (form I) salt.

## 2 Experimental details

The NIC-OXA (form I) salt was prepared by mixing an equimolar ratio of NIC and OXA in Millipore water. Initially, a supersaturation solution was prepared at room temperature and under-saturated by adding water to it. The solution was filtered using Whatman filter paper, and the crystalline product was isolated at room temperature by slow evaporation. NIC-OXA (1:1 or form I) salt was crystallized from water by slow evaporation (Athimoolam and Natarajan, 2007). The prepared salt was analyzed using X-ray powder diffraction (XRPD) and differential scanning calorimetry (DSC) to validate the product material. It crystallizes in the monoclinic *P2*_*1*_ */c* space group with one NIC cation and one oxalate anion (singly deprotonated).

### 2.1 Infrared (IR) spectroscopy

Infrared (IR) measurements were performed using a Bruker Alpha-P ATR-spectrometer equipped with a diamond crystal (Karlsruhe, Germany) in an attenuated total reflection configuration. Data evathluation was carried out with the OPUS program (Bruker, Ettlingen, Germany). A single-beam background without the sample and single-beam spectra of the powered samples in a range of 400–4,000 cm^−1^ were obtained by averaging 20 scans with an optical resolution of 4 cm^−1^.

### 2.2 Raman spectroscopy

Raman spectra were collected using a LabRAM HR 800 Raman spectrometer (Horiba, France) equipped with an Nd:YAG laser operating at 532 nm. Samples were placed on microscope glass slides and were exposed to a laser power of 0.8 mW through an objective lens with ×50 magnification. Typically, 50 scans were accumulated with 1–2 s exposure times from 100 to 4,000 cm^−1^ with a spectral resolution of 2 cm^−1^ for obtaining the spectra.

### 2.3 X-ray powder diffraction (XRPD)

XRPD patterns for the sample were collected using an Empyrean X-ray diffractometer (PANalytical, Netherlands) equipped with a PIXcel detector and a monochromatic Cu–K*α1* radiation X-Ray tube (*λ* = 1.54059 Å). The tube voltage and amperage were set at 45 kV and 40 mA, respectively. The samples were measured on silicon wafer-based zero-background holders. The white product solid sample was scanned in the 2θ range of 5–40°, increasing at a step size of 0.02.

### 2.4 Differential scanning calorimetry (DSC)

DSC analysis of samples was performed on the Q1000 DSC of TA Instruments. Samples (1–2 mg) were crimped in non-hermetic aluminum pans and scanned at a heating rate of 10°C/min under a continuously purged dry nitrogen atmosphere (flow rate 50 mL/min) using a similar empty pan as a reference.

## 3 Quantum chemical computational details

The optimized electronic structures and energies of NIC, OXA, and NIC-OXA salt (monomer and dimer models) were computed by the DFT/B3LYP/6-311++G (d,p) (theory/functional/basis set) methodology (Chengteh et al., 1988; Parr and Yang, 1989; Parr and Yang, 1995) using Gaussian 09 suite (Frisch et al., 2009). The vibrational assignments and wavenumbers of all the normal modes were defined based on Pulay’s recommendation (Fogarasi et al., 1992) using the Gar2Ped program (Martin and Alsenoy, 1995). The obtained wavenumbers were scaled by the wavenumber linear scaling (WLS) procedure to include the anharmonicity effect (Yoshida et al., 2002). The pictorial representation of molecular structures and geometries of calculated data were drawn with GaussView (Frisch et al., 2000). Geometrical and topological parameters of the bonds of interacting atoms at bond critical points (bcps) were studied by Bader’s quantum theory of atoms in molecules (Bader, 1990) using AIMALL program packages (Bader and Cheeseman, 2000). The global reactivity descriptors (hardness (η), softness (S), chemical potential (μ), electrophilicity index (ω), and electronegativity (χ)) were calculated by energies of the highest occupied molecular orbital (HOMO) and lowest unoccupied molecular orbital (LUMO) for all molecules (Pearson, 1963; Pearson, 1989; Lu et al., 2022). The local reactivity descriptors (f_k_, s_k_, and ω_k_) can predict the atoms suitable for nucleophilic, electrophilic, and radical attacks (Flores-Holguín et al., 2019).

## 4 Results and discussion

### 4.1 Geometry optimization and energies

**FIGURE 1:**
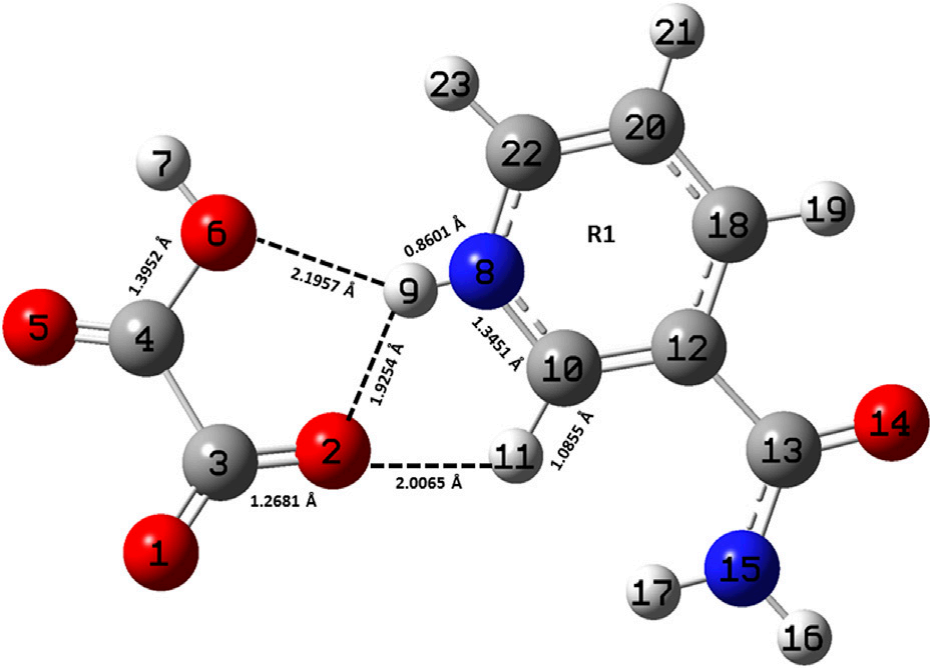
Optimized structure of the monomer model of NIC-OXA (form I) salt with atomic numbering used in the present study.

**FIGURE 2:**
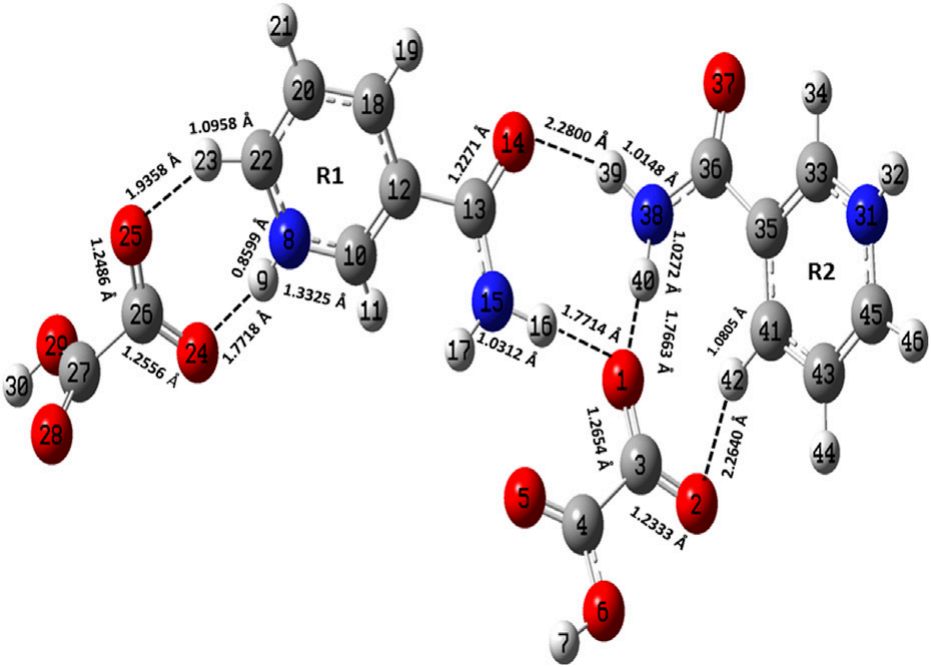
Optimized structure of the dimer model of NIC-OXA (form I) salt with atomic numbering used in the present study.

The structures of NIC, OXA, and NIC-OXA (form I) salt in their crystalline states (Athimoolam and Natarajan, 2007; Jarzembska et al., 2014; Bhattacharya, 2020) are shown in Supplementary Figures S1–S3, respectively, and the optimized ground state structures of NIC, OXA, and monomer and dimer models of NIC-OXA (form I) salt are shown in Supplementary Figures S4, S5; Figures 1, 2, respectively. The optimized ground state geometrical parameters of NIC, OXA, and monomer and dimer models of NIC-OXA (form I) salt along with their experimental values derived from crystalline data are given in Supplementary Table S1. The ground state optimized energies of NIC, OXA, and monomer and dimer models of salt (form I) are −261740.6278, −237480.5176, −499192.8929, and −998362.5581 kcal mol^−1^, respectively.

A comparison was performed between the optimized parameters of NIC and the monomer model of NIC-OXA (form I) salt, which shows that calculations do not differ more than by 0.0030/0.0054 Å, 3.6/2.4°, 1.6/1.0° in experimental/theoretical bond lengths, bond angles, and dihedral angles, respectively, except for the bonds attached with the hydrogen atom. However, the major variations of 0.0044/0.0101 Å in experimental/theoretical values are noticed among the (C10-N8) bond in NIC-OXA (form I) salt, directing the formation of hydrogen bonds: (N8-H9···O2 =C3) and (N8-H9···O6-C4), respectively, between NIC (API) and OXA (coformer) (as shown in Figure 1). The changes in bond angles and dihedral angles of these bonds were found correspondingly.

Similarly, after comparing the experimental/theoretical geometrical parameters of OXA and the monomer model of NIC-OXA (form I) salt, major changes of 0.0406/0.0292 Å, 8.2/6.7° and 2.6/2.0° in bond lengths, bond angles, and dihedral angles, respectively, were found, except for the bonds possessing hydrogen atoms and which are involved in intermolecular hydrogen bonds. A noticeable difference of 0.0862/0.1183 Å was observed in the experimental/optimized bond length parameter of the (O1=C3) bond from OXA to monomer NIC-OXA (form I) salt. This is due to the fact that in individual OXA, a hydrogen atom H8 is attached to O5 of one carboxylic group, while in the NIC-monomer of OXA (form I) salt, this hydrogen was transferred to the N atom of the pyridine ring of NIC, resulting in the (N8-H9) bond. Similarly, the corresponding changes are observed in bond angles and dihedral angles, leading to the presence of intermolecular hydrogen bonds (N8-H9···O2=C3) in the monomer model of salt (form I) (as shown in Figure 1).

The optimized parameters of monomer/dimer models of NIC-OXA (form I) salt (Athimoolam and Natarajan, 2007) were also compared with experimental values (tabulated in Supplementary Table S1). As shown in Supplementary Table S1, the calculated values replicated from experimental results in bond length, bond angle, and dihedral angle by 0.0021/0.0135 Å, 1.1°/1.0°, and 4.5°/0.7° of monomer/dimer in the (C22-N8), (C3-C4=O5), and (C18-C12-C13=O14)/(C12-C18-C20-C22) bonds, respectively, were observed, except for the bonds participating in hydrogen bond formation. However, the differences of 0.0014 Å in (C3=O2) and 0.0076 Å in (C4-O6) bonds were seen due to the presence of intermolecular hydrogen bonds (N8-H9···O6-C4) and (C10-H11···O2=C3), respectively (shown in Figure 1). The theoretical geometrical parameters were in good agreement with those of experimental results in the dimer model of salt (form I) due to the incorporation of the nearest possible hydrogen bond interactions.

It can be seen that the variations in the bond angle of the C–N–C bond from experimental/monomer and dimer models were noticed to be 123.04°/123.29° and 123.60°, respectively, which confirmed the charge transfer phenomenon in NIC-OXA (form I) salt as reported by Allen (2002).

### 4.2 Vibrational assignments and wavenumber

NIC, OXA, and NIC-OXA (monomer/dimer) salt contain 15, 8, and 23/46 atoms, respectively; hence, they possess 39, 18, and 63/132 normal modes of vibrations, active in both IR and Raman spectra. Combined experimental and theoretical vibrational assignments and wavenumbers of two constituents NIC, OXA, and NIC-OXA (monomer/dimer) salt are given in Supplementary Tables S2–S4, respectively. Raman intensities, which are directly proportional to the Raman scattering cross-section, can be derived by Raman scattering amplitudes, as DFT-based calculations do not give these intensities directly (Runge and Gross, 1984; Guirgis et al., 2003). The IR spectrum (related to vibrations) of molecules is generally used to recognize functional groups and chemical bonding in molecular systems. However, defining these vibrational IR bands experimentally is a difficult task and requires theoretical calculation. The temperature and anharmonic effects are generally not considered in computations, and hence discrepancies are noticed between experimental and calculated spectra (Buelna-Garcia et al., 2020; Buelna-García et al., 2021). It has been proposed that the experimental IR spectrum is usually observed at room temperature (293 K) and theoretically simulated at 0 K. So, the discrepancies between observed and calculated wavenumbers of the IR spectrum of NIC-OXA (form I) salt may also be due to such temperature effects.

**FIGURE 3:**
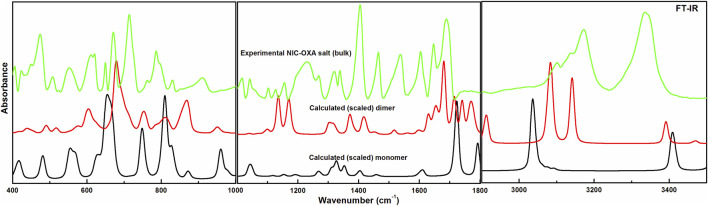
Experimental and calculated (scaled) IR absorbance spectra of NIC-OXA (form I) salt, in the region 400–3,500 cm^−1^.

**FIGURE 4:**
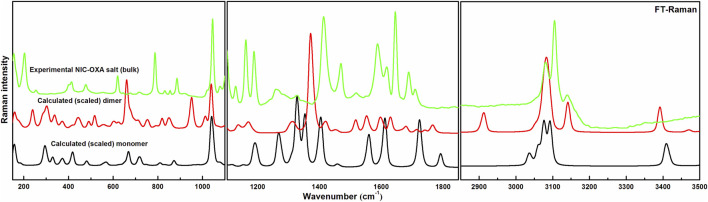
Experimental and calculated (scaled) Raman spectra of NIC-OXA (form I) salt, in the region 100–3,500 cm^−1^.

Comparisons between experimental and calculated IR and Raman spectra for NIC/OXA are shown in Supplementary Figures S6–S9. Comparison between observed IR and Raman spectra of NIC-OXA (form I) salt with that of simulated spectra of monomer/dimer models is depicted in Figures 3, 4, respectively.

Table 1 represents the significant changes in bond lengths and stretching wavenumbers due to the formation of NIC-OXA (form I) salt with its two constituents: NIC and OXA. Figures 1, 2 show that in the monomer/dimer, (one C3=O2 and one C4-O6)/four (C26=O24), (C3=O1), (C26=O25), and (C3=O2) groups of OXA form hydrogen bonds with (N8-H9, C10-H11, and N8-H9)/(N8-H9), (N38-H40)/(N15-H16)/(C41-H42), (C22-H23), and (C41-H42) of NIC, respectively.

**TABLE 1:** Observed and calculated bond-length (Å) and stretching frequency (cm-1) of modes of NIC, OXA, NIC-OXA (form I) salt involved in hydrogen bonding.

Molecules	(C=O) group of NIC				(N-H) of NH2 group of NIC						(C=O) group of OXA			
	Bond-length (Å)	Stretching frequency (cm^-1^)			Bond-length (Å)	Stretching frequency (cm^-1^)					Bond-length (Å)	Stretching frequency (cm^-1^)		
		IR		Raman		IR		Raman				IR		Raman
Experimental														
NIC	1.2373	1615 (C4=O1)		1614 (C4=O1)	1.0103	3357 (N2-H10)		3372 (N2-H10)			-	-		-
					1.0109	3151 (N2-H11)		3153 (N2-H11)			-	-		-
OXA	-	-		-	-	-		-			1.2100	1689 (C2=O3)		1691 (C2=O3)
NIC-OXA (form I) salt	1.2301	1689 (C13=O14)		1688 (C13=O14)	0.8596	3335 (N15-H16, N38-H40)		-			1.2378	1647 (C3=O2, C26=O24)		1646 (C3=O2, C26=O24)
					0.8604	-		3139 (N38-H39)						
Theoretical														
NIC	1.2199	1712 (C4=O1)		1712 (C4=O1)	1.0063	3523 (N2-H10)		3523 (N2-H10)			-	-		-
					1.0087	3410 (N2-H11)		3410 (N2-H11)			-	-		-
OXA	-	-		-	-	-		-			1.1988	1788 (C2=O3)		1788 (C2=O3)
Monomer of salt	1.2183	1726 (C13=O14)		1726 (C13=O14)	1.0088	3520 (N15-H16)		3520 (N15-H16)			1.2681	1722 (C3=O2)		1722 (C3=O2)
					1.0070	3411 (N15-H17)		3411 (N15-H17)						
Dimer of salt	1.2271	1668 (C13=O14)		1668 (C13=O14)	1.0272	3469 (N38-H40)		3469 (N38-H40)			1.2556	1652 (C26=O24)		1652 (C26=O24)
					1.0148	3141 (N38-H39)		3141 (N38-H39)						

#### 4.2.1 Ring vibrations of NIC

The C–N stretching vibrations in aromatic amines are assigned to be in the region of 1,382−1,266 cm^−1^ (Silverstein et al., 1981). In individual NIC, the stretching vibration of (C6-N3) was calculated at 1,272 cm^−1^, corresponding to the observed peaks at 1,253/1,256 cm^−1^ in the IR/Raman spectra, as depicted in Supplementary Table S2. Similarly, in the monomer/dimer model, the same stretching vibrations of (C22-N8) were calculated at 1,116/1,116 cm^−1^, with the IR/Raman peaks at 1,126/1,129 cm^−1^, respectively, as given in Supplementary Table S4. It is clear that C-N in NIC was free from bonding, but in the monomer/dimer model, the protonation occurred with the transfer of hydrogen from OXA to pyridine nitrogen. Hence, a large difference was observed in wavenumbers of NIC and the monomer/dimer. As seen in Table 1, the reduction in calculated stretching wavenumbers of (C-N) bond of 156/156 cm^-1^ with bond length increments of 0.007/0.0095 Å from NIC to monomer/dimer of salt confirms its involvement in intermolecular hydrogen bonds of NIC-OXA (form I) salt. In NIC, the (C6-H12) stretching vibrations were calculated at 3,010 cm^−1^, correspondingly assigned at 3,013/3,010 cm^−1^ in the IR/Raman spectra. The (C-H) in-plane and out-of-plane bending deformation was calculated at 1,341 and 980 cm^−1^, correspondingly observed at 1,340/1,346 and 979/972 cm^−1^, respectively. In the monomer/dimer, the (C10-H11) stretching vibrations were calculated at 3,038/3,056 cm^−1^, which corresponds to the Raman peak at 3,023 cm^−1^. The asymmetric torsion of the ring was calculated at 418 cm^−1^ and observed at 411 cm^−1^ in IR and 415 cm^−1^ in the Raman spectrum region (given in Supplementary Table S4).

#### 4.2.2 Carboxamide (C=O-NH_2_) group vibrations of NIC

NIC consists of a carboxamide group attached to the pyridine ring of the carbon atom. In individual NIC, the stretching vibration of the carbonyl (C4=O1) group was calculated at 1712 cm^−1^. Additionally, the same carbonyl (C13=O14) stretching mode in the monomer/dimer was calculated at 1726/1,668 cm^−1^ and observed experimentally at 1,689/1,688 cm^−1^ in the IR/Raman spectra. It is clear from the aforementioned wavenumber that this carbonyl group was free of bonding in NIC and the monomer; therefore, there is a large difference in the simulated and observed wavenumber values with no reduction in wavenumbers of NIC and the monomer. In the dimer model, there was a reduction of 44 cm^−1^ with an increment in the calculated bond length value of 0.0072 Å. Hence, this group in the dimer was involved in intermolecular hydrogen bonding with neighboring NIC molecules (shown in Figure 2). The stretching vibrations of the (N-H) bond of the amide group are influenced by the presence of hydrogen bonding (Cao et al., 2017). In the assignment of NIC, the stretching vibrations of (N2-H10)/(N2-H11) were calculated at 3,523/3,410 cm^−1^, respectively. In the monomer, the stretching vibrations of (N15-H16) and (N15-H17) modes were calculated at 3,520 and 3,411 cm^−1^, respectively. The stretching vibrations of (N38-H40) and (N15-H16)/(N38-H39) were calculated at 3,469 and 3,392/3,141 cm^−1^ and observed at 3,335 cm^−1^ in the IR spectrum and 3,139 cm^−1^ in the Raman spectrum, respectively. It is clear that NH_2_ group in isolated NIC and monomer were free from bonding. While, in the dimer, the reduction of wavenumbers of 54 and 269 cm^−1^, with increment in bond lengths of 0.0016 and 0.0225 Å, respectively, in (N38-H40) and (N16-H17)/(N38-H39) bonds, showed the presence of intermolecular hydrogen bonding (N38-H40···O1=C3), (N15-H16···O1=C3), and (N38-H39···O14=C13). Therefore, the dimer has values similar to experimental values.

#### 4.2.3 Carboxylic group vibrations of OXA

The stretching vibrations of the (C=O) group lie within the range of 1780–1,680 cm^−1^ (Shreve et al., 1951; Koczón et al., 1999). In the OXA molecule, the stretching vibration of the (C2=O3) group was calculated at 1788 cm^−1^. In the monomer/dimer model, the same stretching vibrations of (C3=O2)/(C26=O24) were calculated to be at 1722/1,652 cm^−1^, corresponding to the peak observed at 1,647/1,646 cm^−1^ in the IR/Raman spectra, which showed that in both monomer/dimer models of salt, there were reductions in calculated wavenumbers of 66/136 cm^−1^ from OXA, along with the increment in bond lengths of 0.0693/0.0568 Å, respectively, hence revealing the presence of intermolecular hydrogen bonds (N8-H9···O2=C3) and (N8-H9···O24=C26), respectively, directing salt formation (as given in Supplementary Table S4). The hydrogen from OXA was transferred from O1 to N8 (pyridine ring) and O1 to N31 and O24 to N8, in the monomer and dimer, respectively. This (C2-O1) bond in individual OXA was calculated to be at 1,127 cm^−1^ and observed at the Raman peak at 1,110 cm^−1^.

### 4.3 X-ray powder diffraction (XRPD)

**FIGURE 5:**
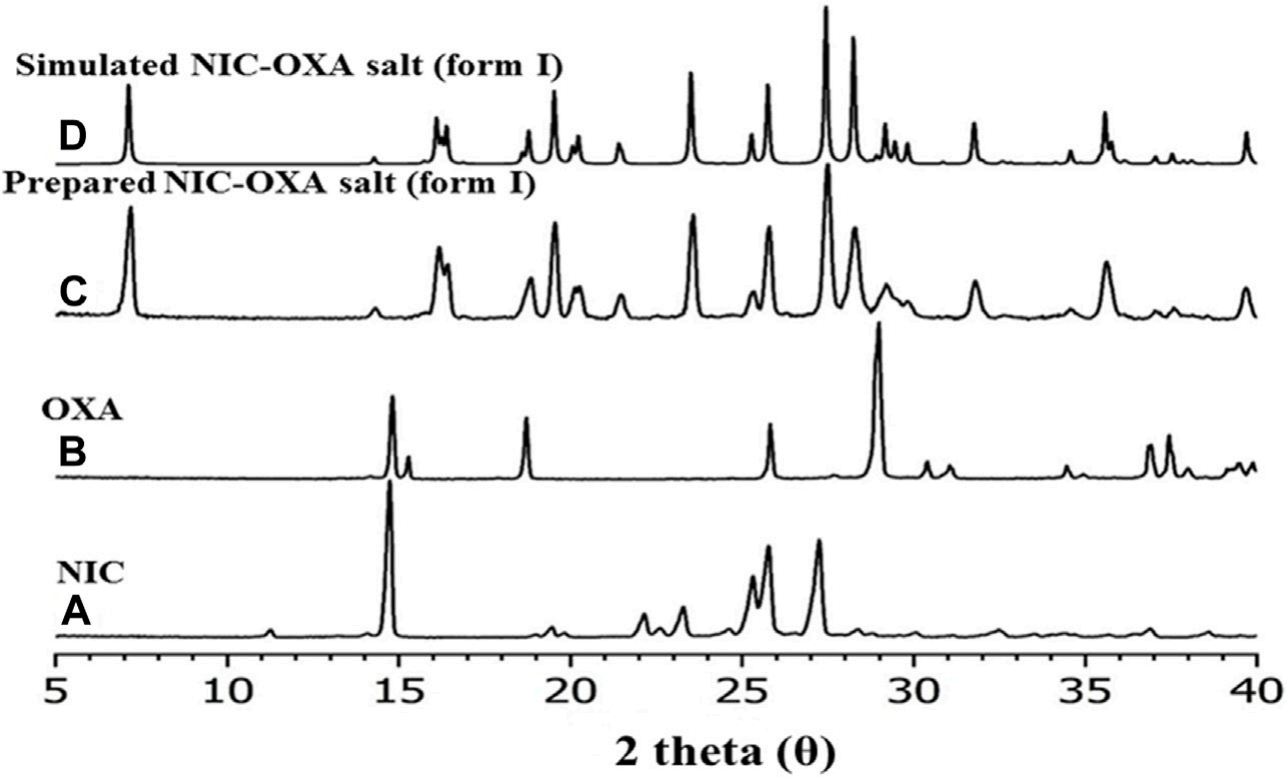
XRPD patterns of **(A)** nicotinamide, **(B)** oxalic acid, **(C)** prepared salt of NIC-OXA (1:1) (form I), and **(D)** simulated single-crystal pattern of NIC-OXA (form I).

The XRPD analysis (Klitou et al., 2020) was performed to identify the phase composition of the isolated material. XRPD data were collected for the initial components (nicotinamide and oxalic acid) and compared with the experimental data (Figure 5C) and the simulated XRPD (Figure 5D) obtained from single-crystal X-ray diffraction. Figure 5 illustrates that the obtained NIC-OXA (form I) salt is found to be in line with the simulated XRPD pattern, and there are no additional peaks present from the initial components. Hence, the isolated material is a single-phase desired material and does not contain unreacted initial components.

### 4.4 Differential scanning calorimetry (DSC)

**FIGURE 6:**
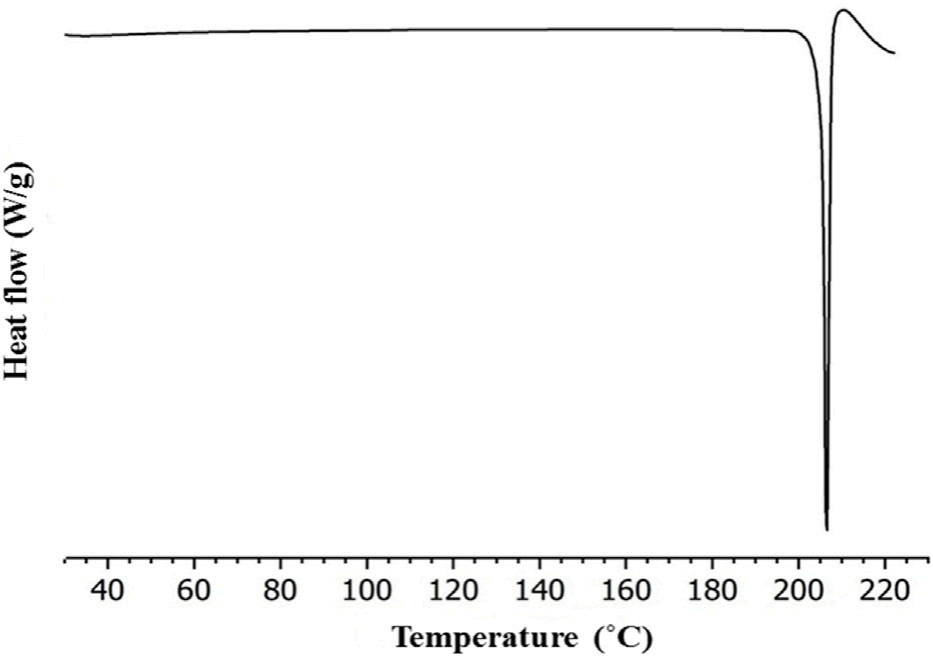
Differential scanning calorimetry (DSC) thermogram of NIC-OXA (form I) salt.

To evaluate the presence of traces of physiosorbed water molecules that are not found XRPD data, differential scanning calorimetry analysis (DSC) was performed. Figure 6 shows the DSC curve (heating rate: 10°C/min under nitrogen as a purge gas). It is evident that the prepared salt has a single endothermic peak at 207°C. There is no other endothermic peak of free water or phase transition observed. Though the materials were prepared from water, there is no trace of physiosorbed water from the DSC thermogram. Figure 6 illustrates the significantly different melting points of NIC-OXA salt compared to the initial components. DSC and XRPD data analysis demonstrate that the prepared NIC-OXA (form I) salt is a single crystalline phase and free from initial components and traces of water molecules (Shimpi et al., 2017).

### 4.5 Topological and energy parameters at the bond critical path (bcp)

Bader’s quantum theory of atoms in molecules (QTAIM) suggests that a bond path between an atomic pair shows an intermolecular hydrogen bonding interaction (Jabłoński, 2015; Jabłoński, 2019). DFT or wavefunction approaches can be used to perform the topological analysis theoretically only by knowledge of the electron density (Tognetti and Joubert, 2014). In QTAIM, the interaction of bonding between two atoms in a molecule is verified by the location of the (3−1) bond critical point (bcp) and the associated bond path (atomic interaction line) between two or more interacting atoms. Geometrical and topological parameters, such as electron density (ρ_b_) and Laplacian of electron density (∇^2^ρ_b_) at bcp, are used to provide the strength and nature of the presence of hydrogen bonds. Koch and Popelier suggested that the presence of hydrogen bonds for (donor proton···acceptor) depends on ρ_b_ within the range of (0.002–0.040 a. u.) and (∇^2^ρ_b_) of 0.024–0.139 a. u. (Koch and Popelier, 1995). Additionally, the criteria by Rozas et al. to describe the hydrogen bond interactions in molecules are as follows: (i) (∇^2^ρ_b_) < 0 and H_b_ < 0 for strong and covalent nature; (ii) (∇^2^ρ_b_) > 0 and H_b_ < 0 for medium and partially covalent nature; and (iii) (∇^2^ρ_b_) > 0 and H_b_ > 0 for weak and electrostatic nature (Alkorta et al., 1998). Furthermore, the concept of strong and weak interactions is associated with conventional and non-conventional hydrogen bonds (Desiraju, 2002).

**FIGURE 7:**
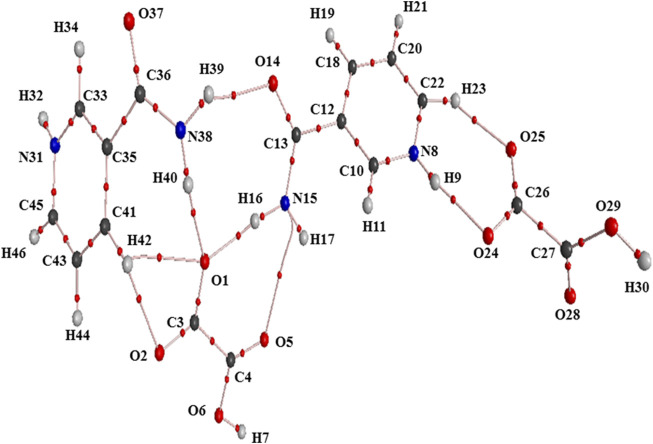
Molecular graph of the dimer model of salt (form I): bond critical points (small red spheres), ring critical points (small yellow sphere), and bond paths (pink lines).

The QTAIM molecular graphs given for the optimized geometries of monomer and dimer models of salt (form I) are given in Supplementary Figure S10; Figure 7, respectively. The geometrical and topological parameters for hydrogen bonds (intramolecular and intermolecular) for monomer and dimer models of salt (form I) are given in Supplementary Table S5; Table 2, respectively.

**TABLE 2:** Geometrical (bond length) and topological parameters for bonds of interacting atoms of intermolecular hydrogen bonding of NIC-OXA (form I) salt: electron density (ρ_b_), Laplacian of electron density (∇^2^ρ_b_), electron kinetic energy density (G_b_), electron potential energy density (V_b_), total electron energy density (H_b_) at the bond critical point (bcp), and estimated interaction energy (E_int_).

Hydrogen bonds	Bond length ( $\overset{{}^{\circ}}{A}$ )	ρ_b_ (a.u.)	∇ ^2^ ρ_b_ (a.u.)	G_b_ (a.u.)	V_b_ (a.u.)	H_b_ (a.u.)	E_int_ (kcal mol^-1^)	G_b_/ρ_b_
N8-H9**···**O24	1.7718	0.041	0.149	0.0006	−0.0385	−0.0379	−12.1	0.0146
N38-H40**···**O1	1.7663	0.037	0.131	0.0001	−0.0331	−0.0329	−10.4	0.0027
N15-H16**···**O1	1.7714	0.038	0.125	0.0008	−0.0330	−0.0322	−10.4	0.0212
C22-H23**···**O25	1.9358	0.029	0.099	−0.0017	−0.0213	−0.0230	−6.7	−0.0576
C41-H42**···**O2	2.2640	0.017	0.057	−0.0019	−0.0107	−0.0125	−3.4	−0.1138
C41-H42**···**O1	2.4123	0.013	0.043	−0.0014	−0.0080	−0.0014	−2.5	−0.1102
N38-H39**···**O14	2.2800	0.013	0.044	−0.0015	−0.0080	−0.0095	−2.5	−0.1154

The criterion for the hydrogen bonds is as follows: the bond distance of two interacting atoms must be smaller than the sum of van der Waals radii of these atoms for the monomer and dimer models of salt (form I), which are listed in Supplementary Tables S6, S7, respectively. In the monomer model of salt (form I), the strongest intermolecular hydrogen bond (conventional) (N8-H9···O2) with interaction energy −8.0006 kcal mol^−1^ possesses medium and partially covalent nature based on the Koch and Popelier criteria. The intermolecular hydrogen bond interaction (C10-H11**···**O2) with energy value −5.8986 kcal mol^−1^ was found to be of the same aforesaid and non-conventional nature. Another hydrogen bond (N8-H9**···**O6) with the calculated interaction energy value of −2.9493 kcal mol^−1^ also possesses a medium (partially covalent) nature (Supplementary Table S5).

In the dimer model of salt (form I), the order of conventional intermolecular hydrogen bonds based on energy values are (N8-H9···O24) > (N38-H40···O1) > (N15-H16···O1) > (N38-H39···O14), which shows the medium and partially covalent nature, while (C22-H23···O25) > (C41-H42···O2) > (C41-H42···O1) are non-conventional intermolecular hydrogen bonds and direct the formation of NIC-OXA (form I) salt (given in Table 2).

### 4.6 Natural bond orbital (NBO) analysis

NBO analysis is a powerful tool to examine the conjugative or hyper-conjugative charge transfer interaction phenomenon and provides a detailed study of intramolecular-and intermolecular hydrogen bonding in a molecular system (Berryman et al., 2020). The electron-donating tendency (i.e., the intensity of charge transfer between electron donor and acceptor) is generally dependent on the extent of stabilization energy E (2) and decides the strength of the bond in a particular molecule. The electron density delocalization between an unoccupied (Rydberg or anti-bonded) and an occupied (lone pair or bonded) orbital corresponds to a stabilized donor–acceptor interaction (Anugrah et al., 2022). The second-order micro-disturbance theory of the donor–acceptor interactions in the NBO basis for monomer and dimer models of NIC-OXA (form I) salt is given in Supplementary Tables S8, S9, respectively.

In the monomer model of salt, the strongest intramolecular hydrogen bond interaction within unit 1 (OXA) unit 2 (NIC) was [LP (3)O2 → π*(O1=C3)] and [LP (1)N15→ π *(C13=O14)] from maximum E^(2)^ values of 82.84 and 63.45 kcal mol^−1^, respectively, which stabilize the molecule and ring R1, respectively. The charge transfer from [unit 1 to unit 2] and [unit 2 to unit 1]; [LP (2)O2 → σ *(N8-H9)] and [σ(C10-H11) → σ *(O2=C3)] with E^(2)^ of 4.50 and 0.25 kcal mol^−1^, respectively, confirms the presence of strong intermolecular hydrogen bonds (N8-H9**···**O2) and (C10-H11**···**O2=C3), respectively (depicted in Supplementary Table S8).

Similarly, in the dimer model of salt, the charge transfer interactions within units 1 (OXA), 2 (NIC), 3 (OXA), and 4 (NIC) [LP (3) O1 → π *(O2=C3)], [LP (1) C12 → π *(N8-C10)], [LP (3) O24 → π *(O25=C26)], and [LP (1) C35 → π *(N31-C33)] with E^(2)^ values of 88.62, 194.72, 101.58, and 297.50 kcal mol^−1^, respectively, confirm the intramolecular charge transfer interactions and stability of NIC-OXA (form I) salt. While other intermolecular charge transfer interactions, such as [LP (1) O1 → σ *(N15-H16)], [LP (1) O1 → σ *(N38-H40)], [σ(N15-H16) → σ *(O1=C3)], [σ(C22-H23) → σ *(O25=C26)], [LP (2) O14 → σ *(N38-H39)], [LP (2) O24 → σ *(N8-H9)], and [σ (N38-H40) → σ *(O1 =C3)], with maximum E^(2)^ values of 10.18, 12.17, 0.18, 0.06, 0.95, 13.44, and 0.36 kcal mol^−1^, respectively, reveal the strong intermolecular hydrogen bonds: (N15-H16**···**O1), (N38-H40**···**O14), (N15-H16**···**O1=C3), (C22-H23**···**O25=C26), (N38-H39**···**O14), (N8-H9**···**O24), and (N38-H40**···**O1=C3), and hence the formation of salt (form I) (depicted in Supplementary Table S9).

### 4.7 Frontier molecular orbitals (FMOs; HOMO–LUMO) analysis

**FIGURE 8:**
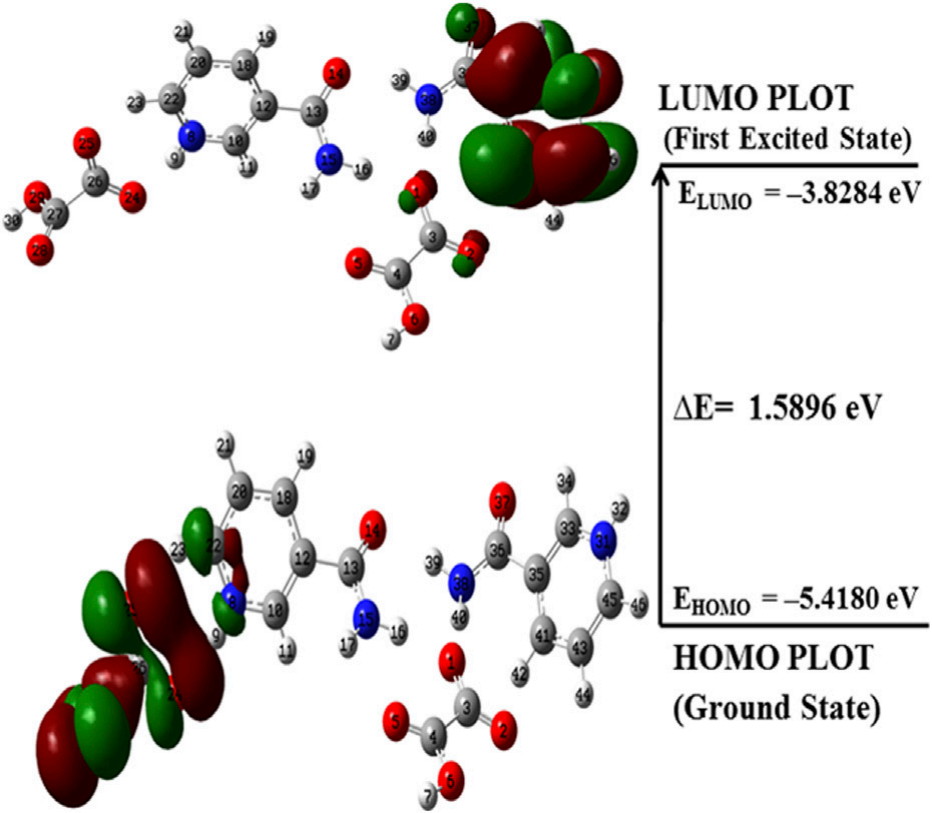
HOMO–LUMO plot of the dimer model of NIC-OXA (form I) salt with the orbital involved in electronic transitions in the isolated (gaseous) phase.

FMOs are widely used to explain the optical and electronic properties of a molecular system (Padmaja et al., 2009). The highest occupied molecular orbital (HOMO) and lowest unoccupied molecular orbital (LUMO) represent the ability to donate and accept electrons, respectively, and their energies correspond to ionization potential (IP) and electron affinity (EA) (Fukui et al., 1952). These orbitals not only explain the charge transfer properties but also molecular chemical reactivity and electrical transport properties (Padmaja et al., 2009). The molecules having a low HOMO–LUMO energy gap are chemically more reactive, have less kinetic stability, and are softer than those with a high energy gap (Aihara, 1999; Ruiz-Morales, 2002; Verma et al., 2021). The HOMO–LUMO plots for NIC, OXA, and monomer and dimer models of salt are shown in Supplementary Figures S11–S13; Figure 8, respectively. Supplementary Figures S11, S13; Figure 8 show that the energy gap values of the NIC (5.5356 eV) > monomer of salt (2.2044 eV) > dimer (1.5896 eV); hence, the salt (form I) was chemically more reactive (less stable) than NIC (API).

### 4.8 Chemical reactivity descriptors

The chemical reactivity descriptors are focused to study the global and local reactivity of any molecule, as discussed in the following section.

#### 4.8.1 Global reactivity descriptors

Global reactivity descriptors are useful to provide insight into the chemical reactivity and stability of chemical entities. Various global reactivity descriptors, such as electronegativity (χ), global hardness (η), global softness (S), chemical potential (μ), and electrophilicity index (ω), can define the global reactivity trends, which are calculated by using energies of frontier molecular orbitals (HOMO and LUMO). The parameter χ defines the electron attracting ability of molecules toward themselves and is an important parameter to provide fruitful information on active sites of an electrophile. According to Pearson, the lowest reactivity of molecules is due to the requirement of a larger resistance value to change its electronic distribution and harder nature (Pearson, 2005). For any molecule, η and S values are inversely proportional to each other. Table 3 reports the global reactivity descriptors for NIC, OXA, and monomer and dimer models of NIC-OXA (salt I).

**TABLE 3:** Calculated E_HOMO_, E_LUMO_, energy band gap (E_L_-E_H_), chemical potential (μ), electro-negativity (χ), global hardness (η), global softness (S), and global electrophilicity index (ω) at 298.15 K for nicotinamide (NIC), oxalic acid (OXA), and NIC-OXA (form I) salt using B3LYP/6-311++G (d, p).

Molecule	E_H_ (eV)	E_L_ (eV)	E_L_-E_H_ (eV)	χ (eV)	μ (eV)	η (eV)	S (eV)	ω (eV)	ΔN_max_
NIC (API)	−7.3414	−1.8058	5.5356	4.5736	−4.5736	2.7678	0.1806	3.7788	1.6524
OXA (coformer)	−8.1669	−2.5002	5.6667	5.3335	−5.3335	2.8333	0.1765	5.0200	1.8824
NIC-OXA salt (monomer)	−6.0205	−3.8161	2.2044	4.9183	−4.9183	1.1022	0.4536	10.9734	4.4622
NIC-OXA salt (dimer)	−5.4180	−3.8284	1.5896	4.6232	−4.6232	0.7948	0.6291	13.4461	5.8168

As shown in Table 3, the value of χ shows that OXA has the highest tendency to attract electrons, so it is favorable for a nucleophilic attack. η values lie in the order of OXA > NIC > monomer of NIC-OXA (form I) salt > dimer of NIC-OXA (form I) salt. It means that the dimer of NIC-OXA (from I) salt is softer than the monomer, NIC, and OXA, and hence, OXA will react easily with NIC. A good electrophile is characterized by higher μ and lower η and *vice versa* for a good nucleophile. Additionally, the stable molecule must have the lowest value of ω. The values of ω lie in the range of NIC < OXA < monomer of NIC-OXA (salt I) < dimer of NIC-OXA (salt I). So, it is clear that NIC is most stable among OXA, monomer, and dimer models of salt (form I).

#### 4.8.2 Local reactivity descriptors

The Fukui function (FF), local softness, and electrophilicity indices are useful tools to infer nucleophilic/electrophilic reactivity trends (Chattaraj and Roy, 2007). Three kinds of condensed Fukui functions ( $\left.f_{k}^{0},f_{k}^{-}{,f}_{k}^{+}\right)$ , local softness ( $s_{k}^{0},s_{k}^{-},s_{k}^{+}$ ), and electrophilicity indices ( $\omega_{k}^{0},\omega_{k}^{-},\omega_{k}^{+}$ ), for radical, electrophilic, and nucleophilic attacks, defined by Hirshfeld atomic charges, distinguished between reactive atomic center species. The values calculated for the monomer and dimer model of salt (form I) are depicted in Supplementary Tables S10, S11, respectively. From Supplementary Table S10, it can be said that in the monomer model of salt (form I), O1 (acts as an electrophile) will be the most preferred site for nucleophilic attacks with the highest ( $\left.f_{k}^{+},s_{k}^{+},\omega_{k}^{+}\right)$ values (0.2864, 0.0536, and 0.5837). Similarly, the C18 (acts as a nucleophile) will be prone to electrophilic attacks with maximum $\left(f_{k}^{-},s_{k}^{-},\omega_{k}^{-}\right)$ values (0.1376, 0.0258, and 0.2805).

From the reported values of Supplementary Table S11, it was observed that for the dimer model of salt (form I), the most reactive sites for nucleophilic and electrophilic attacks are for O25 and C41 atomic sites with ( $f_{k}^{+},s_{k}^{+},\omega_{k}^{+})$ and $\left(f_{k}^{-},s_{k}^{-},\omega_{k}^{-}\right)$ values (0.1404, 0.0263, and 0.2861) and (0.0968, 0.0181, and 0.1974), respectively, showing their participation in intermolecular interactions.

### 4.9 Molecular electrostatic potential [MESP, V(r)] surface

**FIGURE 9:**
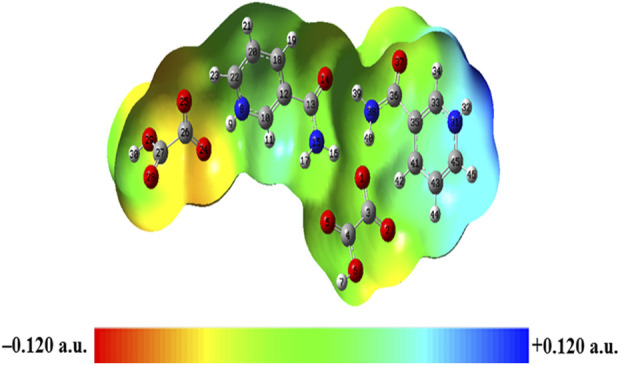
Molecular electrostatic potential (MESP) formed by mapping of total density over electrostatic potential in the gas phase for the dimer model of NIC-OXA (form I) salt.

MESP mapping has been commonly used to predict nucleophilic and electrophilic sites of molecules, plotted by mapping electrostatic potential (ESP) onto the electron density (ED) surfaces, which is taken as constant. The blue (most electropositive potential surface) and red (most electronegative potential region) color represent the electron-accepting and -donating ability of a molecule (Garg et al., 2021). MESP topology analysis determines the understanding of chemical bonding and molecular recognition of active groups in molecular systems (Gadre et al., 2021). The MESP diagrams of NIC and OXA were plotted in a previous study (Verma et al., 2022) (shown in Supplementary Tables S14, S15), which showed the reactive binding sites of two constituents. MESP of NIC displayed negative potential binding sites over the N3 atom of the pyridine ring and positive potential over the–NH_2_ functional group. Additionally, MESP of OXA displayed that positive potential was localized over the hydroxyl (−OH) group, one of which participated in the transfer of a proton from OXA to NIC and negative potential over two carbonyl (C=O) groups in the formation of salt. MESP diagrams of the monomer (Supplementary Figure S16) and dimer model (Figure 9) of NIC-OXA (form I) salt were generated from the deepest red to deepest blue region.

In the monomer model, the oxygen atoms O6 and O2 of OXA were involved in the formation of hydrogen bonds (N8-H9**···**O2 and N8-H9**···**O6), displaying a neutral (green) region (Supplementary Figure S16). The other oxygen (O1 and O5) atoms have been found in the red region and make themselves a site suitable for the nucleophilic attack regions. The -NH_2_ group of NIC was present in the blue region and contributed to probable sites for the electrophilic attack region.

In the dimer model, the MESP surface suggests that (N31-H32) was in the blue region (suitable for nucleophilic attacks) and (C27=O28) was in the red area (susceptible to electrophilic attack), shown in Figure 9. So, these reactive sites are further suitable for forming intermolecular hydrogen bonding with neighboring molecules.

## 5 Conclusion

The molecular structures, geometries, hydrogen bonding interactions, and reactivities of NIC-OXA (form I) salt have been studied by using experimental techniques (IR and Raman) combined with DFT calculations. The structural and vibrational spectroscopic signatures revealed that in the dimer model, (N8-H9) of the pyridine ring of NIC was involved in intermolecular hydrogen bonding with the (C26=O24) group of OXA and possessed a red shifting in wavenumber along with increase in bond length of (C26=O24) of dimer from that of individual OXA. It is found that the vibrational wavenumbers and bond length values of the dimer model of salt match well with the experimental results due to consideration of the nearest possible hydrogen bonding interactions than the monomer. XRPD and DSC analyses suggested that the obtained NIC-OXA (form I) salt has a single-crystalline pure phase. The AIM analysis depicted that the strength of the hydrogen bond (N8-H9**···**O24) is maximum with an interaction energy value −12.1 kcal mol^−1^ that is of medium and partially covalent nature. Furthermore, NBO results showed that the charge transfer interaction from OXA → NIC; [LP (2) O24 → σ *(N8-H9)] is responsible for the stabilization of NIC-OXA (form I) salt. The smaller HOMO–LUMO energy gap of the dimer (1.5896 eV) < monomer (2.2044 eV) < NIC (5.5356 eV) ensured that the chemical reactivity of NIC has been enhanced after formation of NIC-OXA (form I) salt. Hence, the salt is softer and less stable than NIC. The local reactivity parameters defined the most reactive nucleophilic and electrophilic attacking sites for O25 and C41 atoms, respectively, in the dimer model. In the monomer, the nucleophilic and electrophilic reactivities were mainly found around NH_2_ of NIC and (O1 and O5) of OXA, respectively, as viewed from the MESP map. These sites were neutralized in the dimer model, due to the involvement in intermolecular hydrogen bonds. Thus, this study provided the usefulness of combined experimental and theoretical results to define structure-reactivity and hydrogen bonding interactions, which could further be useful to understand other pharmaceutical molecules.

## Data Availability

The original contributions presented in the study are included in the article/Supplementary Material; further inquiries can be directed to the corresponding authors.
