# Supplementary material for: Insights into structural, spectroscopic, and hydrogen bonding interaction patterns of nicotinamide–oxalic acid (form I) salt by using experimental and theoretical approaches
Source: Front Chem. 2023 Jul 5;11:1203278. doi: 10.3389/fchem.2023.1203278 (PMC10354448; doi:10.3389/fchem.2023.1203278)
Supplement: Supplementary file 1 [file DataSheet1.DOCX]

Supplementary Material

**Insights into structural, spectroscopic and hydrogen bonding interactions pattern of nicotinamide-oxalic acid (form I) salt by usingexperimental and theoretical approach**

Priya Verma^1^, Anubha Srivastava^1^, Poonam Tandon^1*^, Manishkumar R. Shimpi^,2*^

^1*^Department of Physics, University of Lucknow, Lucknow, 226007, India.

^2*^Department of Materials and Environmental Chemistry, Stockholm University, Svante Arrhenius väg 16c, 10691 Stockholm, Sweden.

*** Correspondence:**Poonam Tandon
[poonam_tandon@yahoo.co.uk](mailto:poonam_tandon@yahoo.co.uk)

Manishkumar R. Shimpi

[manishshimpi@gmail.com](mailto:manishshimpi@gmail.com%20%20)

# Supplementary Figures and Tables

The crystal structure of NIC, OXA and NIC-OXA (form I) salt with their neighboring interactions are shown in Figure S1-S3. The optimized ground state structure of NIC and OXA are shown in Figures S4 and S5, respectively. Experimental and calculated IR and Raman spectra of NIC and OXA are shown in Figures S6, S7, S8 and S9, respectively. The molecular graph of a monomer model of salt using AIM program is given in Figure S10. HOMO and LUMO plots of NIC, OXA and monomer model of salt with their energy gaps are shown in Figures S11-S13, respectively. The molecular electrostatic potential (MESP) surface of NIC, OXA and monomer model of salt are given in Figures S14, S15 and S16, respectively.

The experimental and calculated geometric parameters of NIC, monomer and dimer models of salt are given in Table S1. Theoretical and experimental vibrational wavenumbers of NIC and OXA are listed in Tables S2 and S3, respectively. The experimental and theoretical wavenumbers of the NIC-OXA saltwith their potential energy distribution (PED) assignments for monomer and dimer models are given in Table S4. Geometrical and topological parameters for intra- and intermolecular hydrogen bonding and geometrical parameters to describe the existence of hydrogen bond interactions for monomer model of salt are given in Tables S5 and S6, respectively. Geometrical parameters obeying the criteria for the existence of hydrogen bonding for dimer model of salt is given in Table S7. Second-order perturbation theory analyses of the Fock Matrix, in the NBO basis for intra- and intermolecular interactions in monomer and dimer models of salt are given in Tables S8 and S9, respectively. Reactivity descriptors as Fukui functions ($f_{k}^{+}{, f}_{k}^{-}$), local softness ($s_{k}^{+}{, s}_{k}^{-})$, local electrophilicity indices ($\omega_{k}^{+}{, \omega}_{k}^{-})$ for monomer and dimer models of NIC-OXA salt using Hirshfeld atomic charges are given in Tables S10 and S11, respectively.

## Supplementary Figures


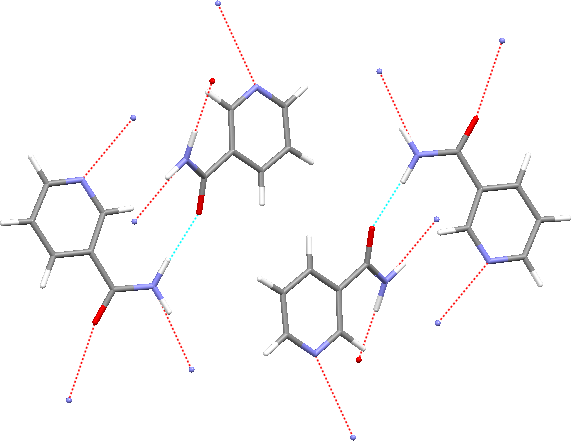


**Figure S1.** The crystal structure of NIC with hydrogen bonding interactionscrystallisesin space group *P21/c* with unit cell parameters *a*=3.8563 Å, *b*=15.6302 Å, *c*=9.3702(3) Å and cell angles*α* = 90˚, *β*= 98.1880˚, *γ*=90˚ (CSD code: NICOAM06).

**
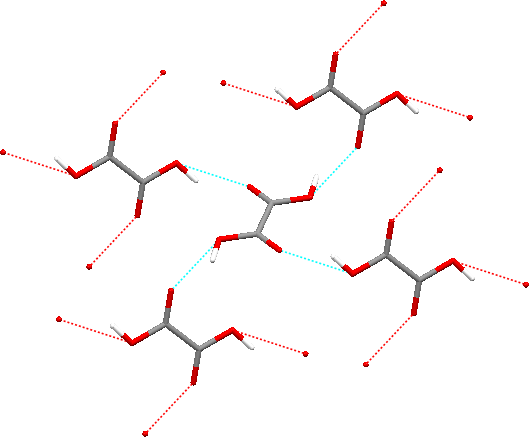
**

**Figure S2** The crystal structure of OXA with space group *Pbca* and unit cell parameters; a=6.4914 Å, b=6.0556 Å, c=7.802 Åand cell angles α = 90˚, β= 90˚, γ=90˚ (CSD code: OXALAC03).


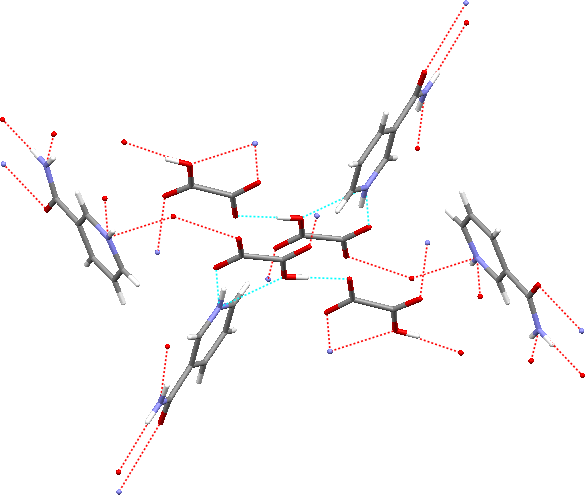


**Figure S3.** Interactions between NIC and OXA molecules which are held together through hydrogen bonds in the crystal lattice of NIC-OXA (form I). The salt crystallises in space group *P21/c*,*a*=12.8295 Å, *b*=6.3148 Å, *c*= 11.1883 Å and cell angles *α* = 90.000˚, *β* = 104.950˚, *γ*=90.000˚ CSD code (LICLEP).


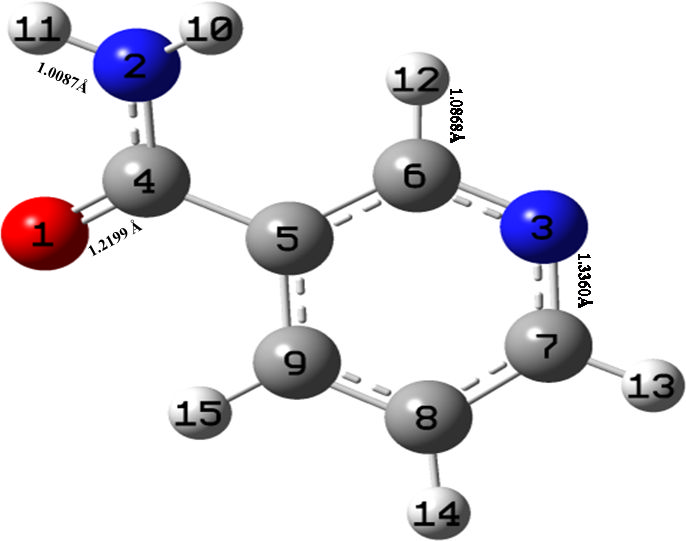


**Figure S4.** Optimized structure of NIC with atomic numbering adopted in this study.


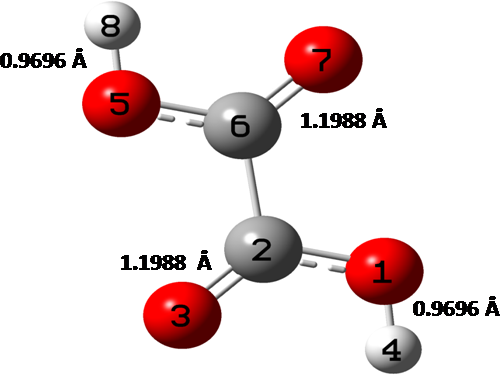


**Figure S5.** Optimized structure of OXA with atomic numbering adopted in this study.



**Figure S6.** Experimental and calculated IR absorbance spectra of NIC in the region 400-3560 cm^−1^.





**Figure S7.**Experimental and calculated Raman scattering spectra of NIC in the region 100-3560 cm^−1^.





**Figure S8.** Experimental and calculated IR absorbance spectra of OXA in the region 400-3850 cm^-1^.





**Figure S9.** Experimental and calculated Raman scattering spectra of OXA in the region 100-3795 cm^-1^.


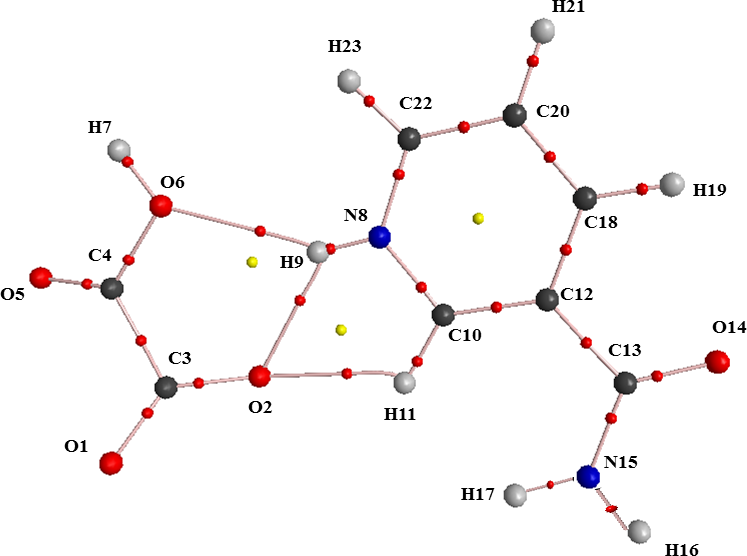


**Figure S10.**Molecular graph of monomer model of NIC-OXA (form I) salt: BCPs (small red spheres), ring critical points (small yellow sphere), bond paths (pink lines).


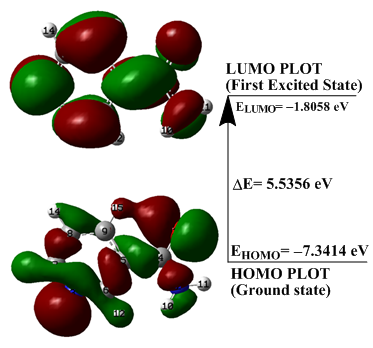


**Figure S11.** HOMO-LUMO plot with their energy gap (ΔE) for NIC with orbital involved in electronic transitions.


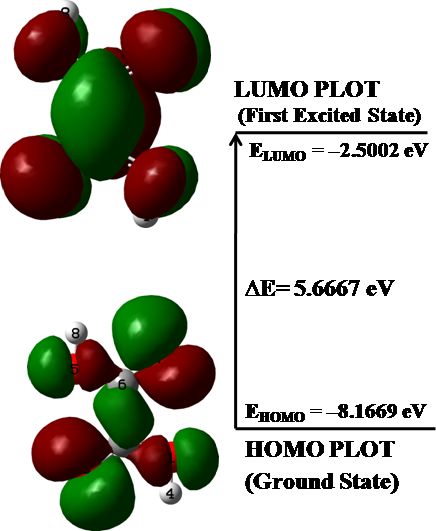


**Figure S12.** HOMO-LUMO plot with their energy gap (ΔE) for OXA with orbital involved in electronic transitions.


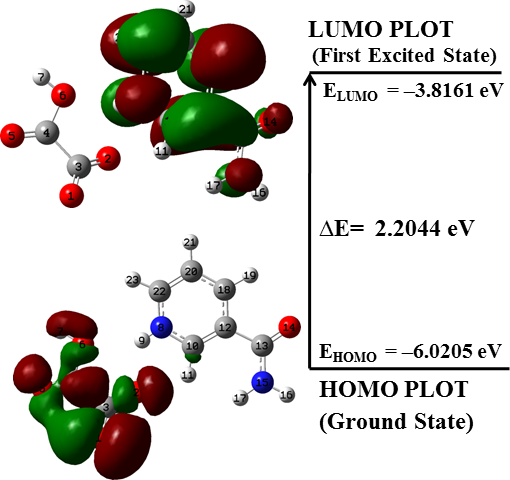


**Figure S13.** HOMO-LUMO plot with their energy gap (ΔE) of monomer of NIC-OXA (form I) salt with orbital involved in electronic transitions.


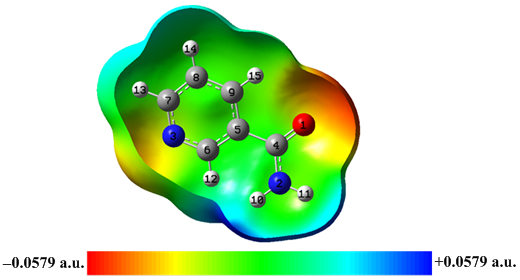


**Figure S14.** Molecular electrostatic potential (MESP) map formed by mapping of total density over electrostatic potential in the gas phase for NIC.


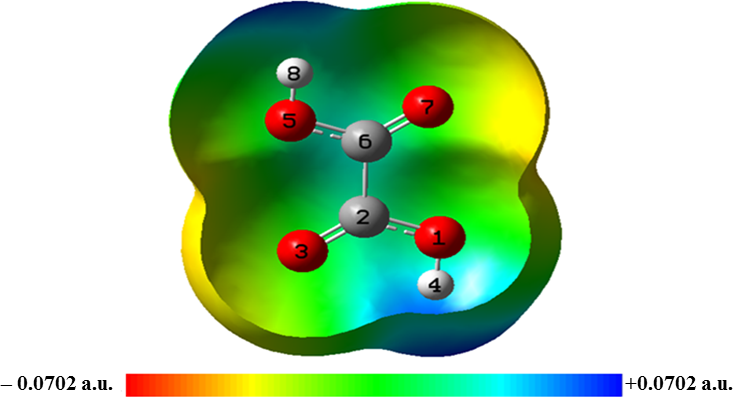


**Figure S15.** Molecular electrostatic potential (MESP) map formed by mapping of total density over electrostatic potential in the gas phase for OXA.


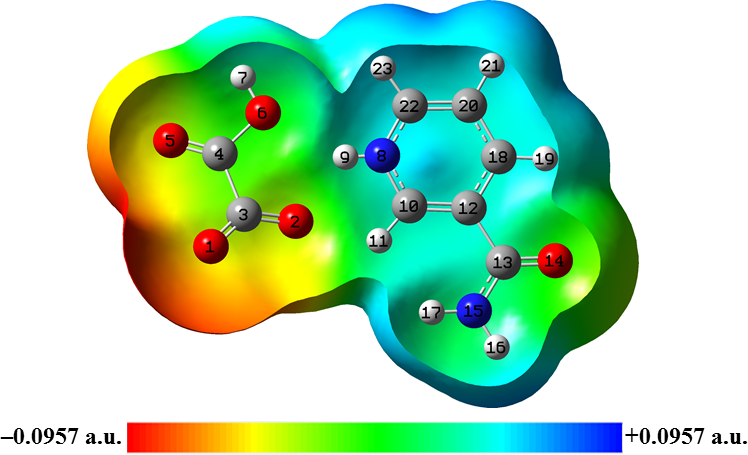


**Figure S16.** Molecular electrostatic potential (MESP) map formed by mapping of total density over electrostatic potential in the gas phase for monomer of NIC-OXA (form I) salt.

## Supplementary Tables

**Table S1.**The experimental geometrical parameters of NIC-OXA (form I) salt and calculated parameters of monomer and dimer models of salt using DFT/6-311++G(d,p), bond lengths in angstroms (Å), bond angles and dihedral angles in degrees (˚).

| **Initial parameters** | **Experimental NIC** | | | | **Optimized NIC** | | **Experimental OXA** | **Optimized OXA** | | | **Experimental NIC-OXA salt (form I)** | | | **Optimized (monomer)** | **Optimized (dimer)** | |
| --- | --- | --- | --- | --- | --- | --- | --- | --- | --- | --- | --- | --- | --- | --- | --- | --- |
| **Bond-length (Å)** | | | | | | | | | | | | | | | | |
| R(O1=C3) | - | | | | - | | 1.3071 | 1.3389 | | | 1.2209 | | 1.2206 | | 1.2654, 1.2556 | |
| R(O2=C3) | - | | | | - | | 1.2100 | 1.1988 | | | 1.2667 | | 1.2681 | | 1.2333, 1.2486 | |
| R(O2-H9) | - | | | | - | | - | - | | | 1.8449 | | 1.9254 | | 2.700, 5.97 | |
| R(O2-H11) | - | | | | - | | - | - | | | 2.1112 | | 2.0065 | | 4.9044, 6.0885 | |
| R(C3-C4) | - | | | | - | | 1.5378 | 1.5443 | | | 1.5784 | | 1.5735 | | 1.5652, 1.5361 | |
| R(C4=O5) | - | | | | - | | 1.2100 | 1.1988 | | | 1.1966 | | 1.1968 | | 1.2084, 1.2057 | |
| R(C4-O6) | - | | | | - | | 1.3071 | 1.3389 | | | 1.4028 | | 1.3952 | | 1.3426, 1.3573 | |
| R(O6-H7) | - | | | | - | | 0.8785 | 0.9696 | | | 0.9704 | | 0.9698 | | 1.0443, 0.9673 | |
| R(O6-H9) | - | | | | - | | - | - | | | 2.3541 | | 2.1957 | | 4.9902, 12.2458 | |
| R(N8-C10) | 1.3406 | | | | 1.3350 | | - | - | | | 1.345 | | 1.3451 | | 1.3325, 1.3489 | |
| R(N8-C22) | 1.3408 | | | | 1.3360 | | - | - | | | 1.3399 | | 1.342 | | 1.3534, 1.3588 | |
| R(C10-H11) | 1.0830 | | | | 1.0868 | | - | - | | | 1.0857 | | 1.0855 | | 1.0791, 1.0807 | |
| R(C10-C12) | 1.3957 | | | | 1.3990 | | - | - | | | 1.3855 | | 1.3855 | | 1.4024, 1.3765 | |
| R(C12-C13) | 1.4980 | | | | 1.5031 | | - | - | | | 1.515 | | 1.5148 | | 1.5081, 1.5245 | |
| R(C12-C18) | 1.3911 | | | | 1.3972 | | - | - | | | 1.3999 | | 1.3988 | | 1.3845, 1.4084 | |
| R(C13=O14) | 1.2373 | | | | 1.2199 | | - | - | | | 1.2187 | | 1.2183 | | 1.2271, 1.2239 | |
| R(C13-N15) | 1.3402 | | | | 1.3690 | | - | - | | | 1.356 | | 1.3555 | | 1.3219, 1.3410 | |
| R(N15-H16) | 1.0109 | | | | 1.0087 | | - | - | | | 1.0102 | | 1.0088 | | 1.0312, 1.0148 | |
| R(N15-H17) | 1.0103 | | | | 1.0063 | | - | - | | | 1.0081 | | 1.007 | | 1.0079, 1.0272 | |
| R(C18-H19) | 1.0834 | | | | 1.0833 | | - | - | | | 1.0844 | | 1.0838 | | 1.0823, 1.0826 | |
| R(C18-C20) | 1.3886 | | | | 1.3879 | | - | - | | | 1.3926 | | 1.3933 | | 1.4035, 1.3968 | |
| R(C20-H21) | 1.0826 | | | | 1.0835 | | - | - | | | 1.0821 | | 1.0815 | | 1.0797, 1.0807 | |
| R(C20-C22) | 1.3912 | | | | 1.3950 | | - | - | | | 1.3849 | | 1.383 | | 1.3716, 1.3776 | |
| R(C22-H23) | 1.0833 | | | | 1.0862 | | - | - | | | 1.0819 | | 1.0811 | | 1.0958, 1.0800 | |
| **Bond-angle (˚)** | | | | | | | | | | | | | | | | |
| A(C3=O2-H9) | | - | | - | | - | | - | | 127.621 | | 120.5148 | | | | 106.2909, 123.6362 |
| A(C3=O2-H11) | | - | | - | | - | | - | | 157.0786 | | 166.1344 | | | | 99.2146, 134.3930 |
| A(H9-O2-H11) | | - | | - | | - | | - | | 64.4179 | | 65.3719 | | | | 54.1151, 10.8453 |
| A(O1=C3=O2) | | - | | - | | 126.9729 | | 125.4462 | | 130.6777 | | 131.0182 | | | | 128.3705, 131.8997 |
| A(O1=C3-C4) | | - | | - | | 110.5319 | | 110.2275 | | 115.1934 | | 115.8474 | | | | 117.7587, 115.8326 |
| A(O2=C3-C4) | | - | | - | | 122.4950 | | 124.3263 | | 114.1289 | | 113.1341 | | | | 113.8620, 112.2676 |
| A(C3-C4=O5) | | - | | - | | 122.4950 | | 124.3263 | | 126.5929 | | 127.6592 | | | | 120.6820, 125.6027 |
| A(C3-C4-O6) | | - | | - | | 110.5319 | | 110.2275 | | 113.9244 | | 112.3631 | | | | 115.5313, 112.4750 |
| A(O5=C4-O6) | | - | | - | | 126.9729 | | 125.4462 | | 119.4784 | | 119.9777 | | | | 123.7782, 121.9222 |
| A(C4-O6-H7) | | - | | - | | 110.5687 | | 107.4579 | | 103.3065 | | 104.8961 | | | | 111.4316, 106.1388 |
| A(C4-O6-H9) | | - | | - | | - | | - | | 105.205 | | 107.8314 | | | | 34.4939, 37.5495 |
| A(H7-O6-H9) | | - | | - | | - | | - | | 150.5609 | | 143.8093 | | | | 125.8954, 68.5898 |
| A(H9-N8-C10) | | - | | - | | - | | - | | 114.7615 | | 115.2457 | | | | 117.3074, 117.6086 |
| A(H9-N8-C22) | | - | | - | | - | | - | | 121.982 | | 121.4585 | | | | 119.11, 120.2105 |
| A(C10-N8-C22) | | - | | - | | - | | - | | 123.242 | | 123.2945 | | | | 123.2110, 122.1788 |
| A(O2-H9-O6) | | - | | - | | - | | - | | 78.1775 | | 80.4205 | | | | 43.0105, 16.3592 |
| A(O2-H9-N8) | | - | | - | | - | | - | | 134.9166 | | 130.1153 | | | | 80.2588, 10.0355 |
| A(O6-H9-N8) | | - | | - | | - | | - | | 137.0629 | | 149.0207 | | | | 109.3080, 7.1303 |
| A(N8-C10-H11) | | - | | - | | - | | - | | 111.8035 | | 111.6191 | | | | 117.2705, 115.2497 |
| A(N8-C10-C12) | | - | | - | | - | | - | | 119.9099 | | 119.7324 | | | | 118.7673, 120.3596 |
| A(H11-C10-C12) | | 121.3578 | | 120.8440 | |  | |  | | 128.2788 | | 128.6479 | | | | 123.9544, 124.3718 |
| A(O2-H11-C10) | | - | | - | | - | | - | | 113.5124 | | 117.5721 | | | | 86.7186, 52.0685 |
| A(C10-C12-C13) | | 124.0754 | | 123.9434 | | - | | - | | 124.0537 | | 123.8656 | | | | 121.8318, 124.6105 |
| A(C10-C12-C18) | | 118.1332 | | 117.7403 | | - | | - | | 118.0982 | | 118.2279 | | | | 118.7736, 118.2831 |
| A(C13-C12-C18) | | 117.7724 | | 118.2981 | | - | | - | | 117.8471 | | 117.9047 | | | | 119.3945, 117.0517 |
| A(C12-C13=O14) | | 119.2676 | | 121.4633 | | - | | - | | 119.4457 | | 119.5779 | | | | 118.7167, 116.7352 |
| A(C12-C13-N15) | | 117.5799 | | 116.4889 | | - | | - | | 117.1959 | | 116.8882 | | | | 116.4390, 118.8512 |
| A(O14=C13-N15) | | 123.1512 | | 122.0416 | | - | | - | | 123.3452 | | 123.5338 | | | | 124.8432, 124.4133 |
| A(C13-N15-H16) | | 117.8411 | | 116.7027 | | - | | - | | 117.6531 | | 117.5393 | | | | 118.6694, 118.0793 |
| A(C13-N15-H17) | | 120.4443 | | 121.9133 | | - | | - | | 124.0343 | | 124.2808 | | | | 123.3035, 122.1142 |
| A(H16-N15-H17) | | 120.3788 | | 117.7892 | | - | | - | | 118.265 | | 118.0758 | | | | 118.0245, 119.0358 |
| A(C12-C18-H19) | | 118.6972 | | 119.0024 | | - | | - | | 118.0438 | | 117.9715 | | | | 118.2473, 118.0172 |
| A(C12-C18-C20) | | 119.5084 | | 118.9235 | | - | | - | | 120.4376 | | 120.4727 | | | | 120.6765, 120.1418 |
| A(H19-C18-C20) | | 121.7871 | | 122.0739 | | - | | - | | 121.5173 | | 121.5558 | | | | 121.0756, 121.8386 |
| A(C18-C20-H21) | | 121.6694 | | 121.1649 | | - | | - | | 121.2605 | | 121.2972 | | | | 121.3736, 120.9071 |
| A(C18-C20-C22) | | 118.0251 | | 118.5593 | | - | | - | | 118.9198 | | 118.856 | | | | 118.7119, 119.6789 |
| A(H21-C20-C22) | | 120.2838 | | 120.2758 | | - | | - | | 119.8193 | | 119.8468 | | | | 119.9126, 119.4129 |
| A(N8-C22-C20) | | 123.4116 | | 123.4454 | | - | | - | | 119.3904 | | 119.4145 | | | | 119.7897, 119.3461 |
| A(N8-C22-H23) | | 116.2234 | | 116.0457 | | - | | - | | 116.2858 | | 116.0223 | | | | 116.1664, 116.5141 |
| A(C20-C22-H23) | | 120.3637 | | 120.5080 | | - | | - | | 124.3226 | | 124.5631 | | | | 124.0438, 124.1377 |
| **Dihedral angle (˚)** | | | | | | | | | | | | | | | | |
| D(H9-O2=C3=O1) | | | - | | - | |  | |  | | -175.773 | | | 156.773 | -88.9654, 161.2205 | |
| D(H9-O2=C3-C4) | | | - | | - | |  | |  | | 4.1487 | | | 23.4702 | 89.9177, -18.6772 | |
| D(H11-O2=C3=O1) | | | - | | - | |  | |  | | -60.7603 | | | -45.1023 | -144.1279, 163.00734 | |
| D(H11-O2=C3-C4) | | | - | | - | |  | |  | | 119.1611 | | | 135.1409 | 34.7551, -16.8904 | |
| D(C3=O2-H9-O6) | | | - | | - | |  | |  | | 1.1941 | | | -10.8985 | -25.9677, 13.4936 | |
| D(C3=O2-H9-N8) | | | - | | - | | - | | - | | 149.1391 | | | 163.2283 | -158.4875, -1.4008 | |
| D(H11-O2-H9-O6) | | | - | | - | | - | | - | | -155.77 | | | 176.717 | 63.7161, -159.7060 | |
| D(H11-O2-H9-N8) | | | - | | - | | - | | - | | -7.8253 | | | -2.5901 | -68.8037, -159.8814 | |
| D(C3=O2-H11-C10) | | | - | | - | | - | | - | | -121.586 | | | 116.0654 | 161.0829, -5.5746 | |
| D(H9-O2-H11-C10) | | | - | | - | | - | | - | | 5.6833 | | | 2.2043 | 57.5862, 2.3557 | |
| D(O1=C3-C4=O5) | | | - | | - | | 0.17400 | | 0.00585 | | -9.5322 | | | -27.0986 | 175.27H23, -163.70337 | |
| D(O1=C3-C4-O6) | | | - | | - | | 180 | | 180 | | 169.7052 | | | 153.0071 | -3.7084, 16.1494 | |
| D(O2=C3-C4=O5) | | | - | | - | | 180 | | 180 | | 170.5338 | | | 152.6975 | -3.7381, 16.2121 | |
| D(O2=C3-C4-O6) | | | - | | - | | -0.17400 | | -0.0058 | | -10.2289 | | | -27.1968 | 177.2812, -163.9351 | |
| D(C3-C4-O6-H7) | | | - | | - | | -175.4514 | | 179.9789 | | -177.535 | | | 178.1439 | -175.0528, 179.9110 | |
| D(C3-C4-O6-H9) | | | - | | - | | - | | - | | 9.8745 | | | 17.5267 | 62.2002, -179.7750 | |
| D(O5=C4-O6-H7) | | | - | | - | | 4.3649 | | -0.0270 | | 1.7612 | | | 1.9527 | 6.0019, -0.2210 | |
| D(O5=C4-O6-H9) | | | - | | - | | - | | - | | -170.829 | | | 162.3766 | -116.7451, 0.0840 | |
| D(C4-O6-H9-O2) | | | - | | - | | - | | - | | -6.5335 | | | -5.6706 | -43.2244, -5.8035 | |
| D(C4-O6-H9-N8) | | | - | | - | | - | | - | | -153.048 | | | 176.9261 | 7.1035, 15.3488 | |
| D(H7-O6-H9-O2) | | | - | | - | | - | | - | | -171.739 | | | 159.4348 | -118.3410, 173.8817 | |
| D(H7-O6-H9-N8) | | | - | | - | | - | | - | | 41.7463 | | | 29.3097 | -68.0132, -164.9659 | |
| D(C10-N8-H9-O2) | | | - | | - | | - | | - | | 7.3644 | | | 2.2605 | 48.4061, 5.8165 | |
| D(C10-N8-H9-O6) | | | - | | - | | - | | - | | 137.6709 | | | 170.956 | 16.2164, -138.5040 | |
| D(C22-N8-H9-O2) | | | - | | - | | - | | - | | -171.292 | | | 177.3322 | -124.8224, -174.7008 | |
| D(C22-N8-H9-O6) | | | - | | - | | - | | - | | -40.9852 | | | -8.6367 | -157.0122, 40.9787 | |
| D(H9-N8-C10-H11) | | | - | | - | | - | | - | | 0.0956 | | | 0.1816 | 3.0526, -1.8672 | |
| D(H9-N8-C10-C12) | | | - | | - | | - | | - | | -178.977 | | | 179.5706 | -175.9735, 179.6405 | |
| D(C22-N8-C10-H11) | | | - | | - | | - | | - | | 178.7327 | | | 179.7659 | 175.9800, 178.6609 | |
| D(C22-N8-C10-C12) | | | - | | - | | - | | - | | -0.34 | | | 0.0137 | -3.0461, 0.1687 | |
| D(H9-N8-C22-C20) | | | - | | - | | - | | - | | 179.0558 | | | 179.8356 | 175.7610, 179.9351 | |
| D(H9-N8-C22-H23) | | | - | | - | | - | | - | | -0.5683 | | | -0.0902 | -4.3019, 0.4455 | |
| D(C10-N8-C22-C20) | | | - | | - | | - | | - | | 0.5149 | | | 0.2764 | 2.9547, -0.6064 | |
| D(C10-N8-C22-H23) | | | - | | - | | - | | - | | -179.109 | | | 179.6494 | -177.1083, 179.9040 | |
| D(N8-C10-H11-O2) | | | - | | - | | - | | - | | -5.0866 | | | -2.1295 | -77.4588, -176.7038 | |
| D(C12-C10-H11-O2) | | | - | | - | | - | | - | | 173.8895 | | | 177.595 | 101.5121, 1.7199 | |
| D(N8-C10-C12-C13) | | | -179.1445 | | -179.62314 | | - | | - | | 179.5281 | | | 179.8849 | -178.9783, 177.9864 | |
| D(N8-C10-C12-C18) | | | - | | - | | - | | - | | -0.0817 | | | -0.3886 | 1.1451, 0.7592 | |
| D(H11-C10-C12-C13) | | | -2.1951 | | -1.4163 | | - | | - | | 0.6248 | | | 0.4101 | 2.0652, -0.3614 | |
| D(H11-C10-C12-C18) | | | - | | - | | - | | - | | -178.985 | | | 179.9064 | -177.8113, -177.5886 | |
| D(C10-C12-C13=O14) | | | 157.646 | | 159.4804 | |  | |  | | 178.7348 | | | 173.7879 | 165.8407, -159.5133 | |
| D(C10-C12-C13-N15) | | | -22.7583 | | -19.6409 | | - | | - | | 0.0127 | | | -6.1241 | -14.3079, 20.2882 | |
| D(C18-C12-C13=O14) | | | - | | - | | - | | - | | -1.6544 | | | -5.7099 | -14.2835, 17.7449 | |
| D(C18-C12-C13-N15) | | | - | | - | | - | | - | | 179.6235 | | | 174.3781 | 165.5679, -162.4535 | |
| D(C10-C12-C18-H19) | | | - | | - | | - | | - | | 179.9058 | | | 179.4867 | -179.5480, 178.1946 | |
| D(C10-C12-C18-C20) | | | - | | - | | - | | - | | 0.3131 | | | 0.48 | 0.7307, -1.2544 | |
| D(C13-C12-C18-H19) | | |  | |  | | - | | - | | 0.2714 | | | 0.04 | 0.57H23, 0.7569 | |
| D(C13-C12-C18-C20) | | | - | | - | | - | | - | | -179.321 | | | 179.9933 | -179.1489, -178.6920 | |
| D(C12-C13-N15-H16) | | | - | | - | | - | | - | | -179.7 | | | 179.5563 | 179.0853, 178.5649 | |
| D(C12-C13-N15-H17) | | | - | | - | | - | | - | | -2.2599 | | | -3.3472 | -1.5153, 8.7237 | |
| D(O14=C13-N15-H16) | | | - | | - | | - | | - | | 1.6321 | | | 0.5356 | -1.0735, -1.6499 | |
| D(O14=C13-N15-H17) | | | - | | - | | - | | - | | 179.0722 | | | 176.7447 | 178.3259, -171.4911 | |
| D(C12-C18-C20-H21) | | | - | | - | | - | | - | | 179.6378 | | | 179.7462 | 178.6492, -179.5452 | |
| D(C12-C18-C20-C22) | | | - | | - | | - | | - | | -0.1444 | | | -0.2015 | -0.8481, 0.8392 | |
| D(H19-C18-C20-H21) | | | - | | - | | - | | - | | 0.0595 | | | -0.2883 | -1.0641, 1.0274 | |
| D(H19-C18-C20-C22) | | | - | | - | | - | | - | | -179.723 | | | 179.764 | 179.4386, -178.5882 | |
| D(C18-C20-C22-N8) | | | - | | - | | - | | - | | -0.2613 | | | -0.1756 | -0.9257, 0.0904 | |
| D(C18-C20-C22-H23) | | | - | | - | | - | | - | | 179.3307 | | | 179.7434 | 179.1425, 179.5386 | |
| D(H21-C20-C22-N8) | | | - | | - | | - | | - | | 179.9534 | | | 179.876 | 179.5694, -179.5310 | |
| D(H21-C20-C22-H23) | | | - | | - | | - | | - | | -0.4547 | | | -0.205 | -0.3624, -0.0828 | |

**Table S2.** Theoretical and experimental vibrational wavenumbers (cm^-1^) of NIC and their assignments using B3LYP/6-311++G(d,p).

| **Scaled**  **(monomeric model)** | **Experimental** | | **Potential Energy**  **Distribution (**$\boldsymbol{\geq5\%}$**)** | **Simplified**  **description of**  **modes** |
| --- | --- | --- | --- | --- |
|  | **IR** | **Raman** |  |  |
| 3523 | 3357 | 3372 | [ν(N2H10)](58)+[ν(N2H11)](41) | N-H stretch |
| 3410 | 3151 | 3153 | [ν(N2H11)](58)+[ν(N2H10)](41) | N-H stretch |
| 3063 | 3060 | 3062 | R1[ν(CH)](99) | Ring CH stretch |
| 3049 | 3047 | 3034 | R1[ν(CH)](99) | Ring CH stretch |
| 3020 | 3019 | 3018 | R1[ν(CH)](97) | Ring CH stretch |
| 3010 | 3013 | 3009 | R1[ν(C6H12)](97) | Ring CH stretch |
| 1712 | 1615 | 1614 | [ν(C4=O1)](71)+[ν(C4N2)](8)+[ρ(C4N2)](6)+[δ_sci_(C4N2)](3) | C=O stretch +  C-N stretch |
| 1600 | 1591 | 1597 | R1[ν(CC)](45)+R1[δ_in_(CH)](17)+R1[ν(C6N3)](15)+R1[δ’_a_](8) | Ring CC stretch |
| 1592 |  |  | [δ_sci_(C4N2)](85)+[ν(C4=O1)](5)+[ν(C4N2)](5) |  |
| 1578 | 1575 | 1579 | R1[ν(CC)](51)+R1[ν(C7N3)](15)+R1[δ_in_(C7H13)](12)+R1[δ_a_](7) | Ring CC stretch |
| 1484 | 1485 | 1491 | R1[δ_in_(CH)](54)+R1[ν(C7N3)](10)+R1[ν(CC)](17) | Ring in plane CH  deformation |
| 1427 | 1421 | 1431 | R1[δ_in_(C7H13)](46)+R1[ν(C6N3)(14)+R1[ν(CC)](26) | Ring in plane CH  deformation |
| 1346 | 1340 | 1346 | [ν(C4N2)](31)+R1[ν(C4C5)](21)+[δ_sym_(C4N2)](12)+[ρ(C4N2)](7)+R1[δ_in_(CH)](11) | C-N stretch |
| 1341 |  |  | R1[δ_in_(CH)](78) | Ring in plane CH  deformation |
| 1272 | 1253 | 1256 | R1[ν(C6N3)](24)+R1[ν(C7N3)](19)+R1[ν(CC)(48) | Ring CN stretch |
| 1213 | 1202 | 1211 | R1[δ_in_(CH)](49)+R1[ν(C7N3)](20)+R1[ν(C7C8)](11)+R1[ν(C6N3)](8) | Ring in plane CH  deformation |
| 1143 | 1153 | 1139 | R1[ν(CC)](36)+R1[δ_in_(C8H14)](22)+R1[δ_tri_](14)+[ν(C4N2)](9)+[ρ(C4N2)](9) | Ring CC stretch |
| 1121 | 1124 | 1124 | R1[δ_in_(CH)](45)+R1[ν(C6N3)](14)+R1[ν(CC)](22) | Ring in plane CH  deformation |
| 1074 | 1067 | 1070 | [ρ(C4N2)](56)+[ν(C4N2)](24)+[ν(C4=O1)](8) | CN rocking |
| 1049 | 1038 | 1042 | R1[ν(CC)](55)+R1[ν(C7N3)](17)+R1[δ_in_(C8H14)](7)+R1[ν(C6N3)](6) | Ring CC stretch |
| 1031 | 1028 | 1034 | R1[δ_tri_](70)+R1[ν(CC)](17) | Ring trigonal  deformation |
| 1006 | 995 | 1005 | R1[oop(CH)](86)+R1[puck](8) | Ring out of plane  CH deformation |
| 980 | 979 | 972 | R1[oop(CH)](90) | Ring out of plane  CH deformation |
| 940 | 935 | 939 | R1[oop(CH)](82)+R1[puck](12) | Ring out of plane  CH deformation |
| 839 | 828 | 834 | R1[oop(CH)](51)+R1[puck](20)+[ω(C4N2)](12)+R1[oop(C4C5)](12) | Ring out of plane  CH deformation |
| 777 | 775 | 777 | R1[δ’_a_](34)+R1[ν(CC)](30)+R1[δ_tri_](7)+[ρ(C4N2)](5)+[ν(C4N2)](5) | Ring asym deformation |
| 748 | 732 | 741 | [ω(C4N2)](51)+R1[oop(CH)](38) | CN wagging |
| 717 | 701 | 705 | R1[puck](79)+R1[oop(C8H14)](13)+R1[oop(C4C5)](5) | Ring puckering |
| 644 | 644 | 646 | R1[δ’_a_](32)+R1[δ_a_](27)+[δ_sym_(C4N2)](18)+[ρ(C4N2)](12) | Ring asym deformation |
| 619 | 620 | 629 | R1[δ_a_](48)+[ρ(C4N2)](23)+[δ_sym_(C4N2)](12) | Ring asym deformation |
| 556 | 550 | 556 | [τ(C4N2)](71)+R1[oop(C4C5)](8)+R1[τ’_a_](5) | CN torsion |
| 497 | 509 | 499 | [ρ(C4N2)](29)+[δ_sym_(C4N2)](17)+R1[δ_in_(C4C5)](12)+R1[oop(C4C5)](8)+R1[τ_a_](7) | CN rocking |
| 418 | 411 | 415 | R1[τ_a_](71)+R1[oop(C4C5)](7) | Ring asym torsion |
| 386 | - | 389 | R1[τ’_a_](63)+R1[oop(C4C5)](17) | Ring asym torsion |
| 378 | - | - | R1[ν(C4C5)](27)+[ρ(C4N2)](24)+R1[δ’_a_](17)+R1[δ_a_](7) | Ring CC stretch |
| 321 | - | 315 | [ω(C4N2)](69)+[τ(C4N2)](17)+[ν(C4N2)](6) | CN wagging |
| 214 | - | 198 | R1[δ_in_(C4C5)](58)+[δ_sym_(C4N2)](13)+[ω(C4N2)](11)+[ρ(C4N2)](5)+[τ(C4N2)](5) | Ring in plane CC  deformation |
| 152 | - | 136 | R1[oop(C4C5)](42)+R1[τ’_a_](26)+[ω(C4N2)](9)+R1[oop(C9H15)](7)+R1[τ_a_](5) | Ring out of plane CC  deformation |
| 55 | - | - | [τ(C4C5)](77)+R1[δ_in_(C4C5)](6)+[ρ(C4N2)](5) | CC torsion |

**Table S3.**Theoretical and experimental vibrational wavenumbers (cm^-1^) of OXA and their assignments using B3LYP/6-311++G(d,p).

| **Unscaled** | **Scaled** | **IR** | **Raman** | **Potential Energy Distribution (**$\boldsymbol{\geq5\%}$**)** |
| --- | --- | --- | --- | --- |
| 3759 | 3562 | 3097 | - | [ν(O1-H4)](50)+[ν(O5-H8)](50) |
| 3759 | 3562 |  | - | [ν(O5-H8)](50)+[ν(O1-H4)](50) |
| 1836 | 1797 |  |  | [ν(C2=O3)](38)+[ν(C6=O7)](38)+[ν(C2-C6)](7)+[ δ (O7=C6-O5)(4)+ δ (O3=C2-C6)(4) |
| 1826 | 1788 | 1689 | 1691 | [ν(C2=O3)] (44)+ [ν(C6=O7)]( 44)+[ν(C6-O5)](4)+ [ν(C2-O1)](4) |
| 1399 | 1379 | 1367 | 1369 | [ν(C2-C6)](19) +[ν(C6-O5)](17)+[ν(C2-O1)](17) +[δsym(C2-C6)]( 10)+[δ(C6H8O5)](10)+[ν(C6-O5)](10)+[δsym(C2-C6)](9)+[ρ(C2-C6)](5) |
| 1324 | 1307 | 1346 | 1340 | [δ(C2H4O1)](31)+[δ(C6H8O5)]( 31)+[ν(C2-O1)]( 10)+[ν(C6-O5)](10) +[δsym(C2-C6)](8)+[ρ(C6-O5)]( 6) |
| 1206 | 1193 | 1161 | 1158 | [δ(C6H8O5)](27)+[δ(C2H4O1)](27)+[ν(C6-O5)](15) +[ν(C2-O1)](15)+[ν(C2-C6)](5) |
| 1138 | 1127 |  | 1110 | [ν(C2-O1)](32) +[ν(C6-O5)](32)+[δ(C6H8O5)](14) +[δ(C2H4O1)](14) |
| 839 | 835 | 868 | 856 | [ω(C6-O5)](49)+[ω(C2-C6)](49) |
| 787 | 784 | 783 | 781 | [ν(C2-C6)](38)+[δsym(C2-C6)](14)+[ρ(C6-O5)](12)+ [ν(C6-O5)](12)+[ν(C2-O1)](12)+[δ(C2H4O1)](5)+ [δ(C6H8O5)](5) |
| 664 | 663 | 675 | 676 | [τ(C6-O5)](40)+[τ(C2-O1)](40)+[ω(C2-C6)](10)+ [ω(C6-O5)](10) |
| 641 | 640 | 652 |  | [δsym(C2-C6)](43)+[ρ(C6-O5)]( 29)+[δsym(C2-C6)]( 11)+[δ(C6H8O5)](7)+[δ(C2H4O1)](7) |
| 607 | 607 |  | 620 | [τ(C2-O1)]( 49)+[τ(C6-O5)]( 49) |
| 529 | 529 | 517 | 522 | [ρ(C2-C6)](39)+[δsym(C2-C6)](30)+[ρ(C6-O5)](13)+[ν(C2-C6)](8)+[ν(C2-O1)](3) +[ν(C6-O5)]( 3) |
| 425 | 426 | 494 | 477 | [ν(C2-C6)](34)+[δsym(C2-C6)]( 30)+[ρ(C6-O5)](26) +[δsym(C2-C6)](3) |
| 419 | 419 | 415 | 397 | [ω(C2-C6)](41)+[ω(C6-O5)](41)+[τ(C6-O5)](9) +[τ(C2-O1)](9) |
| 265 | 266 | - | 281 | [ρ(C2-C6)](48)+[δsym(C2-C6)](36)+[ρ(C6-O5)](16) |
| 5 | 5 | - | - | [τ(C6-O5)](25)+[τ(C2-O1)](25)+[δsym(C2-C6)](14)+ [δsym(C2-C6)](10) +[ρ(C2-C6)](7)+[ω(C6-O5)](6)+ [ω(C2-C6)](6) |

**Table S4** Theoretical and experimental vibrational wavenumbers (cm^-1^) of monomer and dimer of NIC-OXA (form I) salt and their assignments using B3LYP/6-311++G(d,p).

| **Unscaled** | **Scaled (monomer)** | **Scaled (dimer)** | **IR** | **Raman** | **Potential Energy**  **Distribution (**$\boldsymbol{\geq5\%}$**)** |
| --- | --- | --- | --- | --- | --- |
| 3735 | 3540 | 3547, 3528 | - | - | [ν(O6H7](85)+ν[O6H9](10) |
| 3713 | 3520 | -,  3469 | 3335 | - | [ν(N15H17](56)+[ν(N15H16](44) |
| 3589 | 3411 | 3392  3141 | - | 3139 | [ν(N15H16](56)+[ν(N15H17](44) |
| 3236 | 3093 | 3091, 3092 | 3101 | - | R1[ν(C22H23](34)+ϒ[C22N8O6](32)+ϒ[C22N8O2](25) |
| 3217 | 3076 | 3080 | - | 3081 | R1[ν(C20H21](68)+R1[ν(C22H23](9)+ϒ[C22N8O6](8)+R1[ν(C18H19](8)+ϒ[C22N8O2](5) |
| 3200 | 3061 | 3073 | - |  | R1[ν(C18H19](87)+R1[ν(C20H21](9) |
| 3174 | 3038 | 3056 | - | 3023 | R1[ν(C10H11)](97) |
| 1831 | 1792 | 1778 | - | 1710 | [ρ(C4O6)](58)+[ν(C3C4](24) +[ν(C4=O5](8)+[ρ(C3C4)](7) |
| 1762 | 1726 | 1668 | 1689 | 1688 | [ρ(C3C4)](38)+[ν(C13=O14](25) +[ν(C3=O2](7)+[ρ(C4O6)](6)+[ν(C13N15](5) |
| 1757 | 1722 | 1652 | 1647 | 1646 | [ρ(C3C4)](62)+[ν(C3=O2](12)+[ρ(C4O6)](10)+[ν(C3=O1](7)+[δsym(C4O6)](6) |
| 1641 | 1612 | 1631 |  | 1615 | R1[ν(C22N8](32)+ϒ[C22N8O2](23)+R1[δa](10)+[δsci(C13N15](9)+R1[ν(C10C12](7)+ϒ[C22N8O6](5)+2[ν(C3=O1](5) |
| 1630 | 1600 | 1599 | 1604 | 1588 | R1[ν(C22N8](28)+ϒ[C22N8O2](22)+[δsci(C13N15](19)+R1[δa](8) |
| 1586 | 1558 | 1561 | 1560 | 1555 | R1[ν(C22N8](43)+ϒ[C22N8O6](29)+R1[ν(C12H18](7)+R1[ν(C18C20](5) |
| 1483 | 1460 | 1457 | 1466 | 1469 | ϒ[C22N8O6](54)+R1[ν(C22N8](23)+ϒ[C22N8O2](11) |
| 1423 | 1403 | 1419 | 1404 | 1413 | R1[ν(C22N8](40)+ϒ[C22N8O2](30)+ϒ[C22N8O6](6) |
| 1371 | 1352 | 1345 | 1359 | 1345 | R1[ν(C22N8](30)+[ν(C13N15](15)+ϒ[C22N8O6](14)+R1[ν(C12C13](9)+ϒ[C22N8O2](6) |
| 1347 | 1329 | 1326 | 1338 | 1328 | [ν(C3C4](45)+R1[τ’a](22) +[ν(C3=O2](11)+[ρ(C4O6)](9) |
| 1330 | 1313 | 1315 | 1319 |  | ϒ[C22N8O6](27)+R1[ν(C22N8](18)+[ν(C3C4](12)+R1[δin(C10H11)](11)+[ρ(C4O6)](6) |
| 1285 | 1270 | 1307,1302 | 1269 | 1307 | [ρ(C4O6)](21)+ν[O6H9](19)+[δsym(C4O6)](15)+[ρ(C3C4)](11)+R1[τ’a](9)+[ν(C3=O2](8)+ϒ[C22N8O6](7)+[ω(C3C4)](5) |
| 1205 | 1192 | 1165 | 1167 | 1162 | ϒ[C22N8O6](54)+R1[ν(C22N8](30)+ϒ[C22N8O2](5) |
| 1166 | 1154 | 1155 | 1155 | 1143 | ϒ[C22N8O2](51)+ϒ[C22N8O6](10)+R1[δtri](9)+R1[δin(C20H21)](5) |
| 1127 | 1116 | 1116 | 1126 | 1129 | R1[ν(C22N8](43)+ϒ[C22N8O6](37)+R1[δin((C18H19)](4) |
| 1088 | 1079 | 1097 | 1089 | 1081 | [ρ(C13N15](25)+R1[ν(C22N8](22)+ϒ[C22N8O6](19)+[ν(C13N15](12) |
| 1053 | 1044 | 1070 |  |  | [δsym(C4O6)](28) +[ν(C3C4](22)+[ν(C4O6](17)+[ρ(C3C4)](13)+[ρ(C4O6)](10)+ϒ[C22N8O2](5) |
| 1050 | 1041 | 1039 | 1041 | 1045 | ϒ[C22N8O6](54)+ϒ[C22N8O2](33)+R1[ν(C22N8](6) |
| 1036 | 1028 | 1012, 1010 | 1016 | 1023 | R1[oop(C18H19)](52)+R1[oop(C20H21)](20)+R1[oop(C10H11)](8)+R1[puck](8)+R1[τ’a](6) |
| 987 | 980 | 975 | 974 | 979 | R1[oop(C10H11)](57)+R1[puck](11)+R1[oop(C18H19)](9)+R1[δin(C22H23)](5) |
| 980 | 973 | 959 | 962 | 960 | R1[δin(C22H23)](46)+R1[oop(C20H21)](22)+R1[puck](8)+ϒ[C22N8O2](6)+R1[oop(C10H11)](5) |
| 968 | 961 | 951 | 941 | 954 | ϒ[C22N8O2](30)+ϒ[C22N8O6](21)+R1[δtri](20)+R1[δa](10) +R1[ν(C10N8](7) |
| 878 | 873 | 869 | 852 | 854 | ϒ[C22N8O6](60)+R1[ν(C22N8](21)+ϒ[C22N8O2](17) |
| 837 | 833 | 841 | 839 | 830 | [ω(C3C4)](32)+[ω(C4O6)](29)+[ρ(C3C4)](23)+[ρ(C4O6)](8)+[δsym(C4O6)](3) |
| 834 | 829 | 819 | 829 | 821 | R1[oop(C20H21)](27)+R1[δin(C22H23)](24)+R1[puck](14)+R1[oop(C18H19)](10)+[ω(C13N15](10)+R1[oop(C12C13)](9) |
| 812 | 808 | 807,806 | 816 |  | R1[τ’a](52)+[ν(C3C4](32)+ϒ[C22N8O6](5) |
| 753 | 750 | 756,753 | 762 | 751 | [ω(C13N15](49)+R1[oop(C20H21)](13)+R1[oop(C12C13)](11)+R1[oop(C18H19)](8)+R1[δin(C22H23)](6) |
| 720 | 718 | 716 | 713 | 715 | R1[ν(C22N8](54)+ϒ[C22N8O6](29)+ϒ[C22N8O2](7) |
| 671 | 669 | 677 | 671 | 678 | [ρ(C4O6)](43)+ϒ[C22N8O6](21)+ν[O6H9](14)+[δsym(C4O6)](8)+[ν(C3C4](5) |
| 656 | 655 | 661 | 650 |  | [ρ(C4O6)](44)+[ρ(C3C4)](22)+ν[O6H9](11)+ϒ[C22N8O2](6) |
| 640 | 639 | 637 | 636 | 639 | R1[puck](51)+ϒ[C22N8O2](20)+R1[oop(C12C13)](5) |
| 629 | 628 | 624 | 621 | 620 | ϒ[C22N8O2](49) +R1[ν(C22N8](23)+R1[δin(N8H9)](9)+R1[δ’a](5)+R1[δsci(C13N15](5) |
| 567 | 567 | 609 | 611 |  | [ρ(C3C4)](53)+[ρ(C4O6)](15)+ϒ[C22N8O2](11)+[δsym(C4O6)](8)+ϒ[C22N8O6](8) |
| 553 | 553 | 548 | 553 | 553 | τ[C13N15](35)+R1[puck](29)+[ρ(C3C4)](7)+2R1[ν(C10N8](7)+R1[OOP(N8H9)](4) |
| 480 | 480 | 490 | 474 | 477 | ϒ[C22N8O2](41) +R1[ν(C22N8](19)+R1[δa](18)+[ρ(C13N15](8) |
| 416 | 417 | 436 | 449 | 443 | ν[O6H9](34)+[ν(C3C4](24)+ϒ[C22N8O2](21)+[ρ(C4O6)](12) |
| 414 | 415 | 415 | 424 | 414 | 2R1[ν(C10N8](34)+ν[O6H9](15)+R1[oop(C12C13)](13)+[ν(C3C4](9)+ϒ[C22N8O2](8)+[ρ(C4O6)](7) |
| 374 | 375 | 376 | - | - | ϒ[C22N8O2](60)+ϒ[C22N8O6](15)+[ρ(C3C4)](7)+[ρ(C4O6)](5) |
| 369 | 370 | 366 | - | 362 | ϒ[C22N8O2](39)+ϒ[C22N8O6](17)+[ρ(C3C4)](15)+[ρ(C4O6)](13)+ν[O6H9](5) |
| 328 | 329 | 335 | - | 321 | R1[τ’a](48)+R1[OOP(N8H9)](25)+ϒ[C22N8O2](8)+ϒ[C22N8O6](5) |
| 301 | 302 | 307 | - | 303 | [oop(C13N15](15)+R1[δa](14)+R1[δin(N8H9)](8)+R1[τ’a](7)+R1[ν(C22N8](7)+ν[O6H9](6)+[δsym(C4O6)](6)+τ[C13N15](5)+R1[OOP(N8H9)](5) |
| 294 | 295 | 298 | - | 290 | ϒ[C22N8O2](55)+ϒ[C22N8O6](17)+R1[δa](7) |
| 285 | 286 | 283,280 | - | 281 | ν[O6H9](32)+[ρ(C3C4)](26)+ϒ[C22N8O2](25)+[δsym(C4O6)](9)+[ρ(C4O6)](6) |
| 184 | 185 | 187 | - | 204 | ϒ[C22N8O2](61)+ϒ[C22N8O6](34) |
| 158 | 159 | 157 | - | 155 | ϒ[C22N8O2](25)+R1[oop(C12C13)](16)+ϒ[C22N8O6](10)+ν[O6H9](10)+2R1[ν(C10N8](7)+ν[O2H9](6) |
| 153 | 154 | 131 | - | 136 | ϒ[C22N8O2](47)+ν[O6H9](31)+ϒ[C22N8O6](9)+ν[O2H9](6) |
| 70 | 70 | 73,80 | - | - | ν[O6H9](67)+ϒ[C22N8O6](21)+ϒ[C22N8O2](13) |
| 65 | 66 | 65 | - | - | ν[O6H9](66)+ϒ[C22N8O2](18)+ϒ[C22N8O6](14) |
| 47 | 47 | 51 | - | - | ϒ[C22N8O6](41)+ν[O6H9](28)+ϒ[C22N8O2](20)+τ[C3C4](9) |
| 43 | 44 | 44, 45 | - | - | ϒ[C22N8O6](66)+ϒ[C22N8O2](18)+ν[O6H9](10) |
| 42 | 43 | 40 | - | - | ϒ[C22N8O6](37)+ν[O6H9](35)+τ[C3C4](15)+ϒ[C22N8O2](11) |
| 32 | 32 | 30 | - | - | τ[C3C4](75)+ν[O6H9](18) |
| 18 | 18 | 18 | - | - | ϒ[C22N8O2](45)+τ[C3C4](26)+ϒ[C22N8O6](26) |
| 16 | 16 | 15 | - | - | ϒ[C22N8O2](47)+ϒ[C22N8O6](46) |
| 9 | 9 | 9 | - | - | τ[C3C4](59)+ν[O6H9](29)+ϒ[C22N8O2](5) |
| 2 | 2 | 0 | - | - | ϒ[C22N8O6](53)+ϒ[C22N8O2](42) |

**Table S5.** Geometrical parameter (bond length) and topological parameters for bonds of interacting atoms of intra- and intermolecular hydrogen bonding of monomer of NIC-OXA (form I) salt: electron density (ρ_BCP_), Laplacian of electron density (∇^2^ρ_BCP_), electron kinetic energy density (G_BCP_), electron potential energy density (V_BCP_), total electron energy density (H_BCP_) at bond critical point (BCP) and estimated interaction energy (E_int_).

| **Hydrogen bonds** | **Bond-length (Å)** | **ρ_BCP_ (a.u.)** | **∇^2^ρ_BCP_ (a.u.)** | **G_BCP_ (a.u.)** | **V_BCP_**  **(a.u.)** | **H_BCP_ (a.u.)** | **E_int_**  **(kcal mol^-1^)** | **G_BCP_/ ρ_BCP_** |
| --- | --- | --- | --- | --- | --- | --- | --- | --- |
| (N8-H9**···**O2) | 1.9254 | 0.0299 | 0.1309 | -0.0036 | -0.0255 | -0.0292 | -8.0006 | -0.1204 |
| (C10-H11**···**O2) | 2.0065 | 0.0242 | 0.1067 | -0.0039 | -0.0188 | -0.0227 | -5.8986 | -0.1611 |
| (N8-H9**···**O6) | 2.1957 | 0.0135 | 0.0605 | -0.0028 | -0.0094 | -0.0123 | -2.9493 | -0.2074 |

**Table S6.**Geometrical parameters for intermolecular interaction in monomer of NIC-OXA (form I) salt bond distance (Å), bond angle (°) and sum of van der Waals radii of interacting atoms (Å).

| **Interactions (D−H···A)** | ***d*_D−H_** | ***d*_H···A_** | ***d*_D···A_** | **D−H···A** | **(*r*_H_ + *r*_A_)** |
| --- | --- | --- | --- | --- | --- |
| (N8-H9**···**O2) | 0.8601 | 1.9254 | 2.5653 | 130.11 | 2.72 |
| (N8-H9**···**O6) | 0.8601 | 2.1957 | 2.9663 | 149.02 | 2.72 |
| (C10-H11**···**O2) | 1.0855 | 2.0065 | 2.6871 | 117.57 | 2.72 |

**Table S7.**Geometrical parameters for intra- and intermolecular hydrogen bonding interaction in dimer of NIC-OXA (form I) salt: bond distance (Å), bond angle (°) and sum of van der Waals radii of interacting atoms (Å).

| **Interactions (D−H···A)** | ***d*_D−H_** | ***d*_H···A_** | ***d*_D···A_** | **D−H···A** | **(*r*_H_ + *r*_A_)** |
| --- | --- | --- | --- | --- | --- |
| N15-H16…O1 | 1.0312 | 1.7714 | 2.7981 | 173.35 | 2.72 |
| N8-H9…O24 | 0.8599 | 1.7718 | 2.6232 | 170.18 | 2.72 |
| C22-H23…O25 | 1.0958 | 1.9358 | 2.8845 | 142.63 | 2.72 |
| C41-H42…O1 | 1.0805 | 2.4123 | 3.0789 | 118.57 | 2.72 |
| N38-H40…O1 | 1.0272 | 1.7663 | 2.7840 | 170.21 | 2.72 |
| N38-H39…O14 | 1.0148 | 2.2800 | 3.0763 | 134.36 | 2.72 |
| C41-H42…O2 | 1.0805 | 2.2640 | 2.9235 | 117.42 | 2.72 |

**Table S8.** Second-order perturbation theory analyses of the Fock matrix, in the NBO basis for interactions in monomer of NIC-OXA (form I) salt.

| **Donor NBO (i)** | **ED(i)/e** | **Acceptor NBO (j)** | **ED(j)/e** | **E^(2)a^ (kcal/mol)** | **E(j) −E(i)^b^ (a.u.)** | **F(i,j)^c^(a.u.)** |
| --- | --- | --- | --- | --- | --- | --- |
| **within unit 1 (OXA)** | | | | | | |
| LP(2)O1 | 1.84824 | σ*(O2=C3) | 0.06400 | 20.88 | 0.77 | 0.116 |
| LP(2)O1 | 1.84824 | σ*(C3-C4) | 0.15678 | 25.36 | 0.54 | 0.105 |
| LP(2)O2 | 1.89032 | σ*(O1=C3) | 0.03904 | 15.53 | 0.89 | 0.107 |
| LP(2)O2 | 1.89032 | σ *(C3-C4) | 0.15678 | 17.65 | 0.58 | 0.091 |
| LP(3)O2 | 1.66493 | π*(O1=C3) | 0.32102 | 82.84 | 0.29 | 0.138 |
| LP(1)O5 | 1.98049 | σ *(C3-C4) | 0.15678 | 1.72 | 1.03 | 0.039 |
| LP(2)O5 | 1.83062 | σ *(C3-C4) | 0.15678 | 19.78 | 0.60 | 0.098 |
| LP(2)O5 | 1.83062 | σ *(C4-O6) | 0.12474 | 37.40 | 0.56 | 0.131 |
| LP(2)O6 | 1.86038 | π *(C4=O5) | 0.16250 | 33.42 | 0.37 | 0.100 |
| **from unit 1 (OXA) to unit 2 (NIC)** | | | | | | |
| σ(O1=C3) | 1.99439 | σ *(N8-H9) | 0.02979 | 0.06 | 1.76 | 0.009 |
| σ(C3-C4) | 1.97391 | σ *(C10-H11) | 0.02202 | 0.13 | 0.98 | 0.010 |
| LP(1)O2 | 1.96759 | σ *(N8-H9) | 0.02979 | 1.74 | 1.44 | 0.045 |
| LP(1)O2 | 1.96759 | σ *(N8-C10) | 0.02183 | 0.23 | 1.09 | 0.014 |
| LP(1)O2 | 1.96759 | σ *(N8-C22) | 0.02364 | 0.38 | 1.09 | 0.018 |
| LP(1)O2 | 1.96759 | σ *(C10-H11) | 0.02202 | 2.90 | 1.06 | 0.050 |
| LP(1)O2 | 1.96759 | σ *(C10-C12) | 0.02133 | 0.82 | 1.18 | 0.028 |
| LP(2)O2 | 1.89032 | σ *(N8-H9) | 0.02979 | 4.50 | 1.00 | 0.061 |
| LP(2)O2 | 1.89032 | σ *(N8-C22) | 0.02364 | 0.37 | 0.64 | 0.014 |
| LP(2)O2 | 1.89032 | σ *(C10-H11) | 0.02202 | 0.83 | 0.61 | 0.021 |
| LP(3)O2 | 1.66493 | σ *(N8-H9) | 0.02979 | 0.42 | 0.99 | 0.020 |
| LP(3)O2 | 1.66493 | π *(N8-C10) | 0.44867 | 0.08 | 0.12 | 0.003 |
| LP(3)O2 | 1.66493 | σ *(N8-C22) | 0.02364 | 0.08 | 0.64 | 0.007 |
| LP(3)O2 | 1.66493 | σ *(C10-C12) | 0.02133 | 0.06 | 0.73 | 0.006 |
| LP(1)O6 | 1.97919 | σ *(N8-H9) | 0.02979 | 2.07 | 1.41 | 0.048 |
| LP(1)O6 | 1.97919 | σ *(N8-C10) | 0.02183 | 0.12 | 1.05 | 0.010 |
| LP(1)O6 | 1.97919 | σ *C20-C22 | 0.01356 | 0.06 | 1.13 | 0.007 |
| LP(2)O6 | 1.86038 | σ *(N8-H9) | 0.02979 | 0.20 | 1.10 | 0.014 |
| **from unit 2 (NIC) to unit 1 (OXA)** | | | | | | |
| σ(N8-C10) | 1.98493 | σ *(O2=C3) | 0.06400 | 0.05 | 1.48 | 0.008 |
| σ(C10-H11) | 1.97553 | σ *(O2=C3) | 0.06400 | 0.25 | 1.17 | 0.015 |
| **within unit 2 (NIC)** | | | | | | |
| π(N8-C10) | 1.79148 | π *(C12-C18) | 0.29226 | 7.65 | 0.38 | 0.049 |
| π(N8-C10) | 1.79148 | π *C20-C22 | 0.25729 | 23.72 | 0.37 | 0.084 |
| σ(C10-H11) | 1.97553 | σ *(N8-C22) | 0.02364 | 5.77 | 1.01 | 0.068 |
| π(C12-C18) | 1.56603 | π *(N8-C10) | 0.44867 | 45.13 | 0.22 | 0.090 |
| π(C12-C18) | 1.56603 | π *C13=O14 | 0.30108 | 16.02 | 0.30 | 0.064 |
| π(C12-C18) | 1.56603 | π *C20-C22 | 0.25729 | 15.51 | 0.27 | 0.060 |
| π(C20-C22) | 1.62367 | π *(N8-C10) | 0.44867 | 14.16 | 0.24 | 0.053 |
| π(C20-C22) | 1.62367 | π *(C12-C18) | 0.29226 | 23.41 | 0.30 | 0.076 |
| σ(C22-H23) | 1.98139 | σ *(N8-C10) | 0.02183 | 5.29 | 1.03 | 0.066 |
| LP(2)O14 | 1.86272 | σ *(C12-C13) | 0.07693 | 20.46 | 0.64 | 0.103 |
| LP(2)O14 | 1.86272 | σ *(C13-N15) | 0.06278 | 23.99 | 0.72 | 0.119 |
| LP(1)N15 | 1.72351 | π *(C13=O14) | 0.30108 | 63.45 | 0.28 | 0.120 |

**Table S9.** Second-order perturbation theory analysis of the Fock matrix, in the NBO basis for intra- and intermolecular interactions in dimer of NIC-OXA (form I) salt.

| **Donor NBO(i)** | **ED(i)/e** | **Acceptor NBO(j)** | **ED(j)/e** | **E^(2)a^(kcal/mol)** | **E(j)−E(i)^b^(a.u.)** | **F(i,j)^c^(a.u.)** |
| --- | --- | --- | --- | --- | --- | --- |
| **within unit1 (OXA)** | | | | | | |
| LP(2)O1 | 1.87049 | σ*(O2=C3) | 0.04048 | 15.15 | 0.86 | 0.104 |
| LP(2)O1 | 1.87049 | σ*(C3-C4) | 0.14789 | 17.62 | 0.59 | 0.091 |
| LP(3)O1 | 1.65337 | π *(O2=C3) | 0.33993 | 88.62 | 0.28 | 0.140 |
| LP(2)O2 | 1.86146 | σ*(O1=C3) | 0.05814 | 19.42 | 0.79 | 0.113 |
| LP(2)O2 | 1.86146 | σ*(C3-C4) | 0.14789 | 23.48 | 0.57 | 0.103 |
| LP(2)O5 | 1.85558 | σ*(C3-C4) | 0.14789 | 20.40 | 0.60 | 0.099 |
| LP(2)O5 | 1.85558 | σ*(C4-O6) | 0.09114 | 30.43 | 0.64 | 0.126 |
| LP(1)O6 | 1.97772 | σ*(C4=O5) | 0.02078 | 6.32 | 1.25 | 0.079 |
| LP(2)O6 | 1.80446 | π *(C4=O5) | 0.20838 | 48.23 | 0.34 | 0.115 |
| **from unit1 (OXA) to unit2 (NIC)** | | | | | | |
| σ(O1=C3) | 1.99272 | σ*(N15-H16) | 0.04833 | 0.14 | 1.43 | 0.013 |
| σ(O2=C3) | 1.99451 | σ*(N15-H16) | 0.04833 | 0.15 | 1.47 | 0.013 |
| LP(1)O1 | 1.93902 | σ*(N15-H16) | 0.04833 | 10.18 | 1.15 | 0.097 |
| LP(2)O1 | 1.87049 | σ*(C13-N15) | 0.06000 | 0.06 | 0.75 | 0.006 |
| LP(2)O1 | 1.87049 | σ*(N15-H16) | 0.04833 | 8.95 | 0.71 | 0.073 |
| LP(1)O5 | 1.98129 | σ*(C13-N15) | 0.06000 | 0.09 | 1.20 | 0.009 |
| LP(1)O5 | 1.98129 | σ*(N15-H17) | 0.00836 | 0.18 | 1.13 | 0.013 |
| LP(2)O5 | 1.85558 | σ*(C13-N15) | 0.06000 | 0.25 | 0.76 | 0.013 |
| LP(2)O5 | 1.85558 | σ*(N15-H16) | 0.04833 | 0.16 | 0.72 | 0.010 |
| LP(2)O5 | 1.85558 | σ*(N15-H17) | 0.00836 | 0.07 | 0.70 | 0.007 |
| **from unit1 (OXA) to unit4 (NIC)** | | | | | | |
| σ(O1=C3) | 1.99272 | σ*(N38-H40) | 0.04785 | 0.10 | 1.42 | 0.011 |
| π(O2=C3) | 1.98038 | σ*(C41-H42) | 0.01973 | 0.17 | 0.78 | 0.010 |
| π(O2=C3) | 1.98038 | π *(C43-C45) | 0.22883 | 0.08 | 0.30 | 0.005 |
| σ(C3-C4) | 1.97437 | σ*(N38-H40) | 0.04785 | 0.27 | 1.06 | 0.015 |
| σ(C3-C4) | 1.97437 | σ*(C41-H42) | 0.01973 | 0.06 | 1.05 | 0.007 |
| LP(1)O1 | 1.93902 | σ*(N38-H40) | 0.04785 | 12.17 | 1.14 | 0.105 |
| LP(2)O1 | 1.87049 | σ*(N38-H40) | 0.04785 | 5.92 | 0.70 | 0.059 |
| LP(2)O1 | 1.87049 | σ*(C41-H42) | 0.01973 | 0.30 | 0.69 | 0.013 |
| LP(2)O1 | 1.87049 | σ*(C41-C43) | 0.01490 | 0.06 | 0.75 | 0.006 |
| LP(3)O1 | 1.65337 | σ*(N38-H40) | 0.04785 | 0.66 | 0.70 | 0.021 |
| LP(3)O1 | 1.65337 | σ*(C41-H42) | 0.01973 | 0.08 | 0.68 | 0.007 |
| LP(1)O2 | 1.97924 | σ*(C35-C41) | 0.02305 | 0.15 | 1.18 | 0.012 |
| LP(1)O2 | 1.97924 | σ*(C41-H42) | 0.01973 | 0.11 | 1.11 | 0.010 |
| LP(1)O2 | 1.97924 | σ*(C43-H44) | 0.01184 | 0.11 | 1.08 | 0.010 |
| LP(1)O2 | 1.97924 | σ*(C43-C45) | 0.01289 | 0.09 | 1.19 | 0.009 |
| LP(1)O2 | 1.97924 | π *(C43-C45) | 0.22883 | 0.05 | 0.63 | 0.005 |
| LP(2)O2 | 1.86146 | σ*(C35-C41) | 0.02305 | 0.30 | 0.74 | 0.014 |
| LP(2)O2 | 1.86146 | σ*(N38-H40) | 0.04785 | 0.05 | 0.68 | 0.005 |
| LP(2)O2 | 1.86146 | σ*(C41-H42) | 0.01973 | 0.86 | 0.67 | 0.022 |
| LP(2)O2 | 1.86146 | σ*(C43-C45) | 0.01289 | 0.12 | 0.75 | 0.009 |
| LP(2)O2 | 1.86146 | π *(C43-C45) | 0.22883 | 0.14 | 0.19 | 0.005 |
| **from unit2 (NIC) to unit1 (OXA)** | | | | | | |
| σ(N15-H16 | 1.98607 | σ*(O1=C3) | 0.05814 | 0.18 | 1.20 | 0.013 |
| **within unit2 (NIC)** | | | | | | |
| σ(C22-H23) | 1.97736 | σ*(N8-C10) | 0.02420 | 5.87 | 1.01 | 0.069 |
| LP(1)C12 | 0.99976 | π *(N8-C10) | 0.49655) | 194.72 | 0.08 | 0.125 |
| LP(1)C12 | 0.99976 | π *(C13=O14) | 0.32011 | 26.26 | 0.20 | 0.079 |
| LP(1)C12 | 0.99976 | π *(C18-C20) | 0.25444 | 65.97 | 0.14 | 0.109 |
| LP(2)O14 | 1.86687 | σ*(C12-C13) | 0.07437 | 20.49 | 0.64 | 0.104 |
| LP(2)O14 | 1.86687 | σ*(C13-N15) | 0.06000 | 21.03 | 0.75 | 0.114 |
| LP(1)N15 | 1.66668 | π *(C13=O14) | 0.32011 | 57.03 | 0.30 | 0.117 |
| LP*(1)C22 | 0.83209 | π *(N8-C10) | 0.49655) | 89.91 | 0.08 | 0.090 |
| LP*(1)C22 | 0.83209 | π *(C18-C20) | 0.25444 | 49.77 | 0.14 | 0.102 |
| **from unit2 (NIC) to unit3 (OXA)** | | | | | | |
| σ(C22-H23) | 1.97736 | σ*(O25=C26) | 0.05148 | 0.06 | 1.17 | 0.008 |
| **from unit2 (NIC) to unit4 (OXA)** | | | | | | |
| σ(C12-C13) | 1.97323 | σ*(N38-H39) | 0.01494 | 0.06 | 1.09 | 0.007 |
| π(C13=O14) | 1.98351 | σ*(N38-H39) | 0.01494 | 0.23 | 0.85 | 0.012 |
| LP(1)O14 | 1.97691 | σ*(N38-H39) | 0.01494 | 0.74 | 1.12 | 0.026 |
| LP(2)O14 | 1.86687 | σ*(N38-H39) | 0.01494 | 0.95 | 0.69 | 0.024 |
| **from unit3 (OXA) to unit2 (NIC)** | | | | | | |
| σ(O25=C26) | 1.99398 | σ*(C22-H23) | 0.03688 | 0.05 | 1.38 | 0.008 |
| σ(C26-C27) | 1.97408 | σ*(N8-H9) | 0.04722 | 0.27 | 1.40 | 0.017 |
| σ(C26-C27) | 1.97408 | σ*(C22-H23) | 0.03688 | 0.19 | 1.02 | 0.012 |
| LP(1)O24 | 1.97264 | σ*(N8-H9) | 0.04722 | 3.74 | 1.46 | 0.066 |
| LP(2)O24 | 1.85965 | σ*(N8-H9) | 0.04722 | 13.44 | 1.03 | 0.108 |
| LP(2)O24 | 1.85965 | σ*(N8-C10) | 0.02420 | 0.23 | 0.68 | 0.012 |
| LP(2)O24 | 1.85965 | σ*(N8-C22) | 0.02093 | 0.09 | 0.67 | 0.007 |
| LP(3)O24 | 1.61935 | π *(N8-C10) | 0.49655) | 0.29 | 0.12 | 0.005 |
| LP(1)O25 | 1.97155 | σ*(C20-C22) | 0.01485 | 0.06 | 1.17 | 0.007 |
| LP(1)O25 | 1.97155 | σ*(C22-H23) | 0.03688 | 3.88 | 1.07 | 0.058 |
| LP(2)O25 | 1.85754 | σ*(N8-C22) | 0.02093 | 0.10 | 0.66 | 0.007 |
| LP(2)O25 | 1.85754 | σ*(C20-C22) | 0.01485 | 0.36 | 0.74 | 0.015 |
| LP(2)O25 | 1.85754 | σ*(C22-H23) | 0.03688 | 6.87 | 0.64 | 0.061 |
| **within unit3 (OXA)** | | | | | | |
| LP(2)O24 | 1.85965 | σ*(O25=C26) | 0.05148 | 14.38 | 0.84 | 0.100 |
| LP(2)O24 | 1.85965 | σ*(C26-C27) | 0.12839 | 18.43 | 0.62 | 0.096 |
| LP(3)O24 | 1.61935 | π *(O25=C26) | 0.37618 | 101.58 | 0.26 | 0.146 |
| LP(2)O25 | 1.85754 | σ*(O24=C26) | 0.05446 | 15.75 | 0.82 | 0.104 |
| LP(2)O25 | 1.85754 | σ*(C26-C27) | 0.12839 | 19.61 | 0.61 | 0.098 |
| LP(2)O28 | 1.84081 | σ*(C26-C27) | 0.12839 | 19.58 | 0.62 | 0.099 |
| LP(2)O28 | 1.84081 | σ*(C27-O29) | 0.10190 | 32.11 | 0.61 | 0.127 |
| LP(1)O29 | 1.97859 | σ*(C27=O28) | 0.01906 | 6.08 | 1.27 | 0.079 |
| LP(2)O29 | 1.82905 | π *(C27=O28) | 0.20691 | 42.70 | 0.36 | 0.111 |
| **from unit4 (NIC) to unit1 (OXA)** | | | | | | |
| σ(N38-H40) | 1.97814 | σ*(O1=C3) | 0.05814 | 0.36 | 1.20 | 0.019 |
| σ(C41-H42 ) | 1.97665 | σ*(C3-C4) | 0.14789 | 0.06 | 0.90 | 0.007 |
| LP(1)N38 | 1.66421 | σ*(O1=C3) | 0.05814 | 0.07 | 0.83 | 0.007 |
| **within unit4 (OXA)** | | | | | | |
| π(N31-C33) | 1.84395 | LP(1)C35 | 0.97480 | 12.35 | 0.27 | 0.068 |
| π(N31-C33) | 1.84395 | π *(C43-C45) | 0.22883 | 18.83 | 0.40 | 0.079 |
| σ(C33-H34) | 1.97780 | σ*(N31-C45) | 0.02349 | 5.22 | 0.99 | 0.064 |
| π(C36=O37) | 1.97933 | LP(1)C35 | 0.97480 | 6.68 | 0.21 | 0.046 |
| σ(N38-H40) | 1.97814 | σ*(C36=O37) | 0.01559 | 5.77 | 1.24 | 0.076 |
| π(C43-C45) | 1.65486 | LP*(1)C41 | 0.80075 | 47.81 | 0.18 | 0.094 |
| π(C43-C45) | 1.65486 | π *(N31-C33) | 0.51414 | 13.68 | 0.22 | 0.051 |
| LP(1)C35 | 0.97480 | π *(N31-C33) | 0.51414 | 297.50 | 0.05 | 0.129 |
| LP(1)C35 | 0.97480 | π *(C36=O37) | 0.34375 | 35.74 | 0.18 | 0.087 |
| LP(2)O37 | 1.86245 | σ*(C35-C36) | 0.08400 | 22.05 | 0.62 | 0.106 |
| LP(2)O37 | 1.86245 | σ*((C36-N38) | 0.05637 | 21.44 | 0.76 | 0.116 |
| LP(1)N38 | 1.66421 | π *(C36=O37) | 0.34375 | 65.13 | 0.28 | 0.121 |
| LP*(1)C41 | 0.80075 | π *(C43-C45) | 0.22883 | 59.14 | 0.12 | 0.103 |
| π *(N31-C33) | 0.51414 | π *(C43-C45) | 0.22883 | 26.39 | 0.07 | 0.064 |

**Table S10.** Reactivity descriptors as Fukui functions ($f_{k}^{+}{, f}_{k}^{-}$), local softness ($s_{k}^{+}{, s}_{k}^{-})$, local electrophilicity indices ($\omega_{k}^{+}{, \omega}_{k}^{-})$ for monomer of NIC-OXA (form I) salt using Hirshfeld atomic charges.

| **Atom no.** | $\mathbf{f}_{\mathbf{k}}^{\mathbf{+}}$ | $\mathbf{s}_{\mathbf{k}}^{\mathbf{+}}$ | $\boldsymbol{\omega}_{\mathbf{k}}^{\mathbf{+}}$ | $\mathbf{f}_{\mathbf{k}}^{\mathbf{-}}$ | $\mathbf{s}_{\mathbf{k}}^{\mathbf{-}}$ | $\boldsymbol{\omega}_{\mathbf{k}}^{\mathbf{-}}$ |
| --- | --- | --- | --- | --- | --- | --- |
| 1 O | 0.28638 | 0.053641 | 0.583705 | 0.037502 | 0.007024 | 0.076437 |
| 2 O | 0.155774 | 0.029178 | 0.317502 | -0.02213 | -0.00414 | -0.0451 |
| 3 C | 0.10127 | 0.018969 | 0.20641 | 0.010175 | 0.001906 | 0.020739 |
| 4 C | 0.08054 | 0.015086 | 0.164158 | 0.004301 | 0.000806 | 0.008766 |
| 5 O | 0.145949 | 0.027337 | 0.297476 | 0.031194 | 0.005843 | 0.06358 |
| 6 O | 0.072042 | 0.013494 | 0.146837 | -0.01629 | -0.00305 | -0.03321 |
| 7 H | 0.051927 | 0.009726 | 0.105839 | 0.00812 | 0.001521 | 0.01655 |
| 8 N | -0.01699 | -0.00318 | -0.03463 | 0.085144 | 0.015948 | 0.173542 |
| 9 H | -0.00147 | -0.00027 | -0.00299 | 0.023784 | 0.004455 | 0.048477 |
| 10 C | -0.007 | -0.00131 | -0.01428 | 0.058145 | 0.010891 | 0.118512 |
| 11 H | -0.00058 | -0.00011 | -0.00118 | 0.027934 | 0.005232 | 0.056936 |
| 12 C | 0.012183 | 0.002282 | 0.024832 | 0.071516 | 0.013395 | 0.145765 |
| 13 C | 0.005376 | 0.001007 | 0.010957 | 0.035084 | 0.006571 | 0.071509 |
| 14 O | 0.030728 | 0.005756 | 0.06263 | 0.058616 | 0.010979 | 0.119472 |
| 15 N | 0.003893 | 0.000729 | 0.007935 | 0.035503 | 0.00665 | 0.072363 |
| 16 H | 0.010263 | 0.001922 | 0.020918 | 0.03494 | 0.006545 | 0.071215 |
| 17 H | -0.00448 | -0.00084 | -0.00913 | 0.018546 | 0.003474 | 0.037801 |
| 18 C | 0.020467 | 0.003834 | 0.041716 | 0.13764 | 0.025781 | 0.28054 |
| 19 H | 0.01375 | 0.002575 | 0.028026 | 0.058912 | 0.011035 | 0.120076 |
| 20 C | 0.02002 | 0.00375 | 0.040805 | 0.06395 | 0.011978 | 0.130344 |
| 21 H | 0.014431 | 0.002703 | 0.029414 | 0.042257 | 0.007915 | 0.086129 |
| 22 C | 0.002769 | 0.000519 | 0.005644 | 0.133877 | 0.025076 | 0.272871 |
| 23 H | 0.002714 | 0.000508 | 0.005532 | 0.061251 | 0.011473 | 0.124843 |

**Table S11.** Reactivity descriptors as Fukui functions ($f_{k}^{+}{, f}_{k}^{-}$), local softness ($s_{k}^{+}{, s}_{k}^{-})$, local electrophilicity indices ($\omega_{k}^{+}{, \omega}_{k}^{-})$ for dimer of NIC-OXA (form I) salt using Hirshfeld atomic charges.

.

| **Atom no.** | $\mathbf{f}_{\mathbf{k}}^{\mathbf{+}}$ | $\mathbf{s}_{\mathbf{k}}^{\mathbf{+}}$ | $\boldsymbol{\omega}_{\mathbf{k}}^{\mathbf{+}}$ | $\mathbf{f}_{\mathbf{k}}^{\mathbf{-}}$ | $\mathbf{s}_{\mathbf{k}}^{\mathbf{-}}$ | $\boldsymbol{\omega}_{\mathbf{k}}^{\mathbf{-}}$ |
| --- | --- | --- | --- | --- | --- | --- |
| 1 O | 0.030875 | 0.005783 | 0.06293 | -0.00325 | -0.00061 | -0.00663 |
| 2 O | 0.100959 | 0.01891 | 0.205777 | 0.002041 | 0.000382 | 0.00416 |
| 3 C | 0.028087 | 0.005261 | 0.057247 | 0.006044 | 0.001132 | 0.012319 |
| 4 C | 0.023099 | 0.004327 | 0.047081 | 0.005546 | 0.001039 | 0.011304 |
| 5 O | 0.046007 | 0.008617 | 0.093772 | 0.008906 | 0.001668 | 0.018152 |
| 6 O | 0.025124 | 0.004706 | 0.051208 | 0.009197 | 0.001723 | 0.018746 |
| 7 H | 0.01652 | 0.003094 | 0.033671 | 0.011498 | 0.002154 | 0.023435 |
| 8 N | -0.00735 | -0.00138 | -0.01499 | 0.032673 | 0.00612 | 0.066595 |
| 9 H | 0.006045 | 0.001132 | 0.012321 | 0.007743 | 0.00145 | 0.015782 |
| 10 C | 0.003087 | 0.000578 | 0.006292 | 0.029867 | 0.005594 | 0.060875 |
| 11 H | 0.002408 | 0.000451 | 0.004908 | 0.012104 | 0.002267 | 0.024671 |
| 12 C | 0.009925 | 0.001859 | 0.020229 | 0.0136 | 0.002547 | 0.02772 |
| 13 C | 0.004429 | 0.00083 | 0.009027 | 0.004679 | 0.000876 | 0.009537 |
| 14 O | 0.017553 | 0.003288 | 0.035777 | 0.004892 | 0.000916 | 0.009971 |
| 15 N | 0.000117 | 2.19E-05 | 0.000238 | 0.004704 | 0.000881 | 0.009588 |
| 16 H | 0.002648 | 0.000496 | 0.005397 | 0.001744 | 0.000327 | 0.003555 |
| 17 H | 0.000199 | 3.73E-05 | 0.000406 | 0.006682 | 0.001252 | 0.013619 |
| 18 C | 0.016443 | 0.00308 | 0.033514 | 0.044528 | 0.00834 | 0.090758 |
| 19 H | 0.011428 | 0.002141 | 0.023293 | 0.018782 | 0.003518 | 0.038282 |
| 20 C | 0.013511 | 0.002531 | 0.027538 | 0.028243 | 0.00529 | 0.057565 |
| 21 H | 0.010948 | 0.002051 | 0.022314 | 0.017681 | 0.003312 | 0.036038 |
| 22 C | 0.000902 | 0.000169 | 0.001838 | 0.038609 | 0.007232 | 0.078694 |
| 23 H | 0.004069 | 0.000762 | 0.008294 | 0.014278 | 0.002674 | 0.029102 |
| 24 O | 0.094491 | 0.017699 | 0.192593 | -0.0031 | -0.00058 | -0.00632 |
| 25 O | 0.140377 | 0.026294 | 0.286119 | 0.001603 | 0.0003 | 0.003267 |
| 26 C | 0.052221 | 0.009781 | 0.106438 | 0.003484 | 0.000653 | 0.007101 |
| 27 C | 0.056233 | 0.010533 | 0.114615 | 0.003308 | 0.00062 | 0.006742 |
| 28 O | 0.13416 | 0.025129 | 0.273447 | 0.009362 | 0.001754 | 0.019082 |
| 29 O | 0.056442 | 0.010572 | 0.115041 | 0.003703 | 0.000694 | 0.007548 |
| 30 H | 0.038204 | 0.007156 | 0.077868 | 0.006996 | 0.00131 | 0.014259 |
| 31 N | 0.009057 | 0.001696 | 0.01846 | 0.06679 | 0.01251 | 0.136133 |
| 32 H | 0.005589 | 0.001047 | 0.011392 | 0.031748 | 0.005947 | 0.064709 |
| 33 C | 0.008246 | 0.001545 | 0.016807 | 0.066885 | 0.012528 | 0.136326 |
| 34 H | 0.005945 | 0.001114 | 0.012117 | 0.032627 | 0.006111 | 0.066501 |
| 35 C | 0.001323 | 0.000248 | 0.002697 | 0.039545 | 0.007407 | 0.080601 |
| 36 C | 0.003717 | 0.000696 | 0.007576 | 0.011776 | 0.002206 | 0.024002 |
| 37 O | 0.016143 | 0.003024 | 0.032903 | 0.037036 | 0.006937 | 0.075487 |
| 38 N | -0.00218 | -0.00041 | -0.00445 | 0.015856 | 0.00297 | 0.032318 |
| 39 H | 0.001004 | 0.000188 | 0.002046 | 0.014829 | 0.002778 | 0.030225 |
| 40 H | -0.00117 | -0.00022 | -0.00239 | 0.00269 | 0.000504 | 0.005483 |
| 41 C | -0.00478 | -0.00089 | -0.00974 | 0.096844 | 0.01814 | 0.197389 |
| 42 H | -0.00209 | -0.00039 | -0.00425 | 0.032076 | 0.006008 | 0.065378 |
| 43 C | 0.000208 | 3.9E-05 | 0.000424 | 0.045072 | 0.008442 | 0.091867 |
| 44 H | 0.00091 | 0.00017 | 0.001855 | 0.028383 | 0.005316 | 0.057851 |
| 45 C | 0.011284 | 0.002114 | 0.022999 | 0.087703 | 0.016427 | 0.178758 |
| 46 H | 0.007597 | 0.001423 | 0.015484 | 0.043948 | 0.008232 | 0.089576 |
